# Genetic variation across RNA metabolism and cell death gene networks is implicated in the semantic variant of primary progressive aphasia

**DOI:** 10.1038/s41598-019-46415-1

**Published:** 2019-07-26

**Authors:** Luke W. Bonham, Natasha Z. R. Steele, Celeste M. Karch, Iris Broce, Ethan G. Geier, Natalie L. Wen, Parastoo Momeni, John Hardy, Zachary A. Miller, Maria Luisa Gorno-Tempini, Christopher P. Hess, Patrick Lewis, Bruce L. Miller, William W. Seeley, Claudia Manzoni, Rahul S. Desikan, Sergio E. Baranzini, Raffaele Ferrari, Jennifer S. Yokoyama, International FTD-Genomics Consortium (IFGC), D. G. Hernandez, M. A. Nalls, J. D. Rohrer, A. Ramasamy, J. B. J. Kwok, C. Dobson-Stone, P. R. Schofield, G. M. Halliday, J. R. Hodges, O. Piguet, L. Bartley, E. Thompson, E. Haan, I. Hernández, A. Ruiz, M. Boada, B. Borroni, A. Padovani, C. Cruchaga, N. J. Cairns, L. Benussi, G. Binetti, R. Ghidoni, G. Forloni, D. Albani, D. Galimberti, C. Fenoglio, M. Serpente, E. Scarpini, J. Clarimón, A. Lleó, R. Blesa, M. Landqvist Waldö, K. Nilsson, C. Nilsson, I. R. A. Mackenzie, G. -Y. R. Hsiung, D. M. A. Mann, J. Grafman, C. M. Morris, J. Attems, T. D. Griffiths, I. G. McKeith, A. J. Thomas, P. Pietrini, E. D. Huey, E. M. Wassermann, A. Baborie, E. Jaros, M. C. Tierney, P. Pastor, C. Razquin, S. Ortega-Cubero, E. Alonso, R. Perneczky, J. Diehl-Schmid, P. Alexopoulos, A. Kurz, I. Rainero, E. Rubino, L. Pinessi, E. Rogaeva, P. St George-Hyslop, G. Rossi, F. Tagliavini, G. Giaccone, J. B. Rowe, J. C. M. Schlachetzki, J. Uphill, J. Collinge, S. Mead, A. Danek, V. M. Van Deerlin, M. Grossman, J. Q. Trojanowski, J. van der Zee, M. Cruts, C. Van Broeckhoven, S. F. Cappa, I. Leber, D. Hannequin, V. Golfier, M. Vercelletto, A. Brice, B. Nacmias, S. Sorbi, S. Bagnoli, I. Piaceri, J. E. Nielsen, L. E. Hjermind, M. Riemenschneider, M. Mayhaus, B. Ibach, G. Gasparoni, S. Pichler, W. Gu, M. N. Rossor, N. C. Fox, J. D. Warren, M. G. Spillantini, H. R. Morris, P. Rizzu, P. Heutink, J. S. Snowden, S. Rollinson, A. Richardson, A. Gerhard, A. C. Bruni, R. Maletta, F. Frangipane, C. Cupidi, L. Bernardi, M. Anfossi, M. Gallo, M. E. Conidi, N. Smirne, R. Rademakers, M. Baker, D. W. Dickson, N. R. Graff-Radford, R. C. Petersen, D. Knopman, K. A. Josephs, B. F. Boeve, J. E. Parisi, A. M. Karydas, H. Rosen, J. C. van Swieten, E. G. P. Dopper, H. Seelaar, Y. A. L. Pijnenburg, P. Scheltens, G. Logroscino, R. Capozzo, V. Novelli, A. A. Puca, M. Franceschi, A. Postiglione, G. Milan, P. Sorrentino, M. Kristiansen, H. -H. Chiang, C. Graff, F. Pasquier, A. Rollin, V. Deramecourt, T. Lebouvier, D. Kapogiannis, L. Ferrucci, S. Pickering-Brown, A. B. Singleton

**Affiliations:** 1 Memory and Aging Center, Department of Neurology, University of California, San Francisco, San Francisco, CA USA; 2 Neuroradiology Section, Department of Radiology and Biomedical Imaging, University of California, San Francisco, San Francisco, CA USA; 3 Department of Psychiatry, Washington University, St. Louis, MO USA; 4 Texas Tech University Health Science Center, Laboratory of Neurogenetics, Lubbock, TX USA; 5 Department of Molecular Neuroscience, UCL Institute of Neurology, London, UK; 6 School of Pharmacy, University of Reading, Whiteknights, Reading, UK; 7 Department of Neurology, University of California, San Francisco, San Francisco, CA USA; 8 Laboratory of Neurogenetics, National Institute on Aging, National Institutes of Health, Bethesda, MD USA; 9 Reta Lila Weston Research Laboratories, Department of Molecular Neuroscience, UCL Institute of Neurology, London, UK; 10 Dementia Research Centre, Department of Neurodegenerative Disease, UCL Institute of Neurology, London, UK; 11 Department of Medical and Molecular Genetics, King’s College London Tower Wing, Guy’s Hospital, London, UK; 12 The Jenner Institute, University of Oxford, Oxford, UK; 13 Neuroscience Research Australia, Sydney, NSW Australia; 14 School of Medical Sciences, University of New South Wales, Sydney, NSW Australia; 15 South Australian Clinical Genetics Service, SA Pathology (at Women’s and Children’s Hospital), North Adelaide, SA Australia; 16 Department of Paediatrics, University of Adelaide, Adelaide, SA Australia; 17 Research Center and Memory Clinic of Fundació ACE, Institut Català de Neurociències Aplicades, Barcelona, Spain; 18 Neurology Clinic, University of Brescia, Brescia, Italy; 19 Department of Psychiatry, Washington University, St. Louis, MO USA; 20 Hope Center, Washington University School of Medicine, St. Louis, MO USA; 21 Hope Center, Washington University School of Medicine, St. Louis, MO USA; 22 Department of Pathology and Immunology, Washington University, St. Louis, MO USA; 23 Molecular Markers Laboratory, IRCCS Istituto Centro San Giovanni di Dio Fatebenefratelli, Brescia, Italy; 24 MAC Memory Clinic, IRCCS Istituto Centro San Giovanni di Dio Fatebenefratelli, Brescia, Italy; 25 Biology of Neurodegenerative Disorders, IRCCS Istituto di Ricerche Farmacologiche “Mario Negri”, Milano, Italy; 26 University of Milan, Milan, Italy; 27 Fondazione Cà Granda, IRCCS Ospedale Maggiore Policlinico, Milan, Italy; 28 Memory Unit, Neurology Department and Sant Pau Biomedical Research Institute, Hospital de la Santa Creu i Sant Pau, Universitat Autònoma de Barcelona, Barcelona, Spain; 29 Center for Networker Biomedical Research in Neurodegenerative Diseases (CIBERNED), Madrid, Spain; 30 Unit of Geriatric Psychiatry, Department of Clinical Sciences, Lund University, Lund, Sweden; 31 Clinical Memory Research Unit, Department of Clinical Sciences, Lund University, Lund, Sweden; 32 Department of Pathology and Laboratory Medicine, University of British Columbia, Vancouver, Canada; 33 Division of Neurology, University of British Columbia, Vancouver, Canada; 34 Institute of Brain, Behaviour and Mental Health, University of Manchester, Salford Royal Hospital, Salford, UK; 35 Departments of Physical Medicine and Rehabilitation, Psychiatry, and Cognitive Neurology & Alzheimer’s Disease Center, Rehabilitation Institute of Chicago, Chicago, USA; 36 Feinberg School of Medicine, Northwestern University, Chicago, USA; 37 Department of Psychology, Weinberg College of Arts and Sciences, Northwestern University, Chicago, USA; 38 Newcastle Brain Tissue Resource, Institute for Ageing, Newcastle University, Newcastle upon Tyne, UK; 39 Institute of Neuroscience and Institute for Ageing, Campus for Ageing and Vitality, Newcastle University, Newcastle upon Tyne, UK; 40 Institute of Neuroscience, Newcastle University Medical School, Newcastle upon Tyne, UK; 41 IMT School for Advanced Studies, Lucca, Lucca, Italy; 42 Taub Institute, Departments of Psychiatry and Neurology, Columbia University, New York, NY USA; 43 Behavioral Neurology Unit, National Insititute of Neurological Disorders and Stroke, National Insititutes of Health, Bethesda, MD USA; 44 Department of Laboratory Medicine & Pathology, University of Alberta Edmonton, Alberta, Canada; 45 Neurogenetics Laboratory, Division of Neurosciences, Center for Applied Medical Research, Universidad de Navarra, Pamplona, Spain; 46 Department of Neurology, Clínica Universidad de Navarra, University of Navarra School of Medicine, Pamplona, Spain; 47 Neuroepidemiology and Ageing Research Unit, School of Public Health, Faculty of Medicine, The Imperial College of Science, Technology and Medicine, London, UK; 48 West London Cognitive Disorders Treatment and Research Unit, West London Mental Health Trust, London, UK; 49 Department of Psychiatry and Psychotherapy, Technische Universität München, Munich, Germany; 50 Neurology I, Department of Neuroscience, University of Torino, Italy, A.O. Città della Salute e della Scienza di Torino, Torino, Italy; 51 Tanz Centre for Research in Neurodegenerative Diseases, University of Toronto, Toronto, Ontario Canada; 52 Cambridge Institute for Medical Research, and the Department of Clinical Neurosciences, University of Cambridge, Cambridge, UK; 53 Division of Neurology and Neuropathology, Fondazione IRCCS Istituto Neurologico Carlo Besta, Milano, Italy; 54 Department of Clinical Neurosciences, John Van Geest Brain Repair Centre, Forvie Site, University of Cambridge, Cambridge, UK; 55 MRC Cognition and Brain Sciences Unit, Cambridge, UK; 56 Behavioural and Clinical Neuroscience Institute, Cambridge, UK; 57 University of California San Diego, Department of Cellular & Molecular Medicine, La Jolla, CA USA; 58 MRC Prion Unit, Department of Neurodegenerative Disease, UCL Institute of Neurology, Queen Square House, Queen Square, London, UK; 59 Neurologische Klinik und Poliklinik, Ludwig-Maximilians-Universität, Munich, Germany; 60 Department of Pathology and Laboratory Medicine, University of Pennsylvania Perelman School of Medicine, Philadelphia, PA USA; 61 Department of Neurology and Penn Frontotemporal Degeneration Center, University of Pennsylvania Perelman School of Medicine, Philadelphia, PA USA; 62 Neurodegenerative Brain Diseases group, Department of Molecular Genetics, VIB, Antwerp, Belgium; 63 Laboratory of Neurogenetics, Institute Born-Bunge, University of Antwerp, Antwerp, Belgium; 64 Neurorehabilitation Unit, Department of Clinical Neuroscience, Vita-Salute University and San Raffaele Scientific Institute, Milan, Italy; 65 CRICM; UPMC Univ Paris 06, UMR_S975, Paris, France; 66 UPMC Univ Paris 06, UMR_S975, Université Pierre et Marie Curie, Paris, France; 67 AP-HP, Département de neurologie-centre de références des démences rares, Hôpital de la Salpêtrière, Paris, France; 68 CNRS UMR 7225, F-75013, Paris, France; 69 Service de Neurologie, Inserm U1079, CNR-MAJ, Rouen University Hospital, Rouen, France; 70 Service de Neurologie, CH Saint Brieuc, Rouen, France; 71 Service de neurologie, CHU de Nantes, Nantes, France; 72 Department of Neurosciences, Psychology, Drug Research and Child Health (NEUROFARBA) University of Florence, Florence, Italy; 73 Danish Dementia Research Centre, Neurogenetics Clinic, Department of Neurology, Rigshospitalet, Copenhagen University Hospital, Copenhagen, Denmark; 74 Department of Cellular and Molecular Medicine, Section of Neurogenetics, The Panum Institute, University of Copenhagen, Copenhagen, Denmark; 75 Department for Psychiatry & Psychotherapy, Saarland University Hospital, Homburg, Saar Germany; 76 Laboratory for Neurogenetics, Saarland University, Homburg, Saar Germany; 77 Department of Psychiatry, Psychotherapy and Psychosomatics, University Regensburg, Regensburg, Germany; 78 Luxembourg Centre For Systems Biomedicine (LCSB), University of Luxembourg, Esch-sur-Alzette, Luxembourg; 79 Tübingen Site, German Center for Neurodegenerative Diseases, Tübingen, Germany; 80 Salford Royal Foundation Trust, Faculty of Medical and Human Sciences, University of Manchester, Manchester, UK; 81 Regional Neurogenetic Centre, ASPCZ, Lamezia Terme, Italy; 82 Department of Neuroscience, Mayo Clinic Jacksonville, Jacksonville, FL USA; 83 Department of Neurology, Mayo Clinic Rochester, Rochester, MN USA; 84 Department of Pathology, Mayo Clinic Rochester, Rochester, MN USA; 85 Department of Neurology, Erasmus Medical Centre, Rotterdam, The Netherlands; 86 Department of Medical Genetics, VU university Medical Centre, Amsterdam, The Netherlands; 87 Alzheimer Centre and Department of Neurology, VU University Medical Centre, Amsterdam, The Netherlands; 88 Department of Basic Medical Sciences, Neurosciences and Sense Organs, “Aldo Moro” University of Bari, Bari, Italy; 89 Medical Genetics Unit, Fondazione Policlinico Universitario A. Gemelli, Rome, Italy; 90 Cardiovascular Research Unit, IRCCS Multimedica, Milan, Italy; 91 Department of Medicine and Surgery, University of Salerno, Baronissi, SA Italy; 92 Neurology Department, IRCCS Multimedica, Milan, Italy; 93 Department of Clinical Medicine and Surgery, University of Naples Federico II, Naples, Italy; 94 Geriatric Center Frullone ASL Napoli 1 Centro, Naples, Italy; 95 UCL Genomics, Institute of Child Health (ICH), UCL, London, UK; 96 Dept NVS, Alzheimer Research Center, Karolinska Institutet, Stockholm, Sweden; 97 Dept of Geriatric Medicine, Genetics Unit, Karolinska University Hospital, Stockholm, Sweden; 98 Université des Sciences et Technologies de Lille, Inserm 1171, DISTALZ, CHU 59000, Lille, France; 99 National Institute on Aging (NIA/NIH), Baltimore, MD USA; 100 Clinical Research Branch, National Institute on Aging, Baltimore, MD USA

**Keywords:** Gene expression, Genetic association study, Dementia, Neurodegenerative diseases

## Abstract

The semantic variant of primary progressive aphasia (svPPA) is a clinical syndrome characterized by neurodegeneration and progressive loss of semantic knowledge. Unlike many other forms of frontotemporal lobar degeneration (FTLD), svPPA has a highly consistent underlying pathology composed of TDP-43 (a regulator of RNA and DNA transcription metabolism). Previous genetic studies of svPPA are limited by small sample sizes and a paucity of common risk variants. Despite this, svPPA’s relatively homogenous clinicopathologic phenotype makes it an ideal investigative model to examine genetic processes that may drive neurodegenerative disease. In this study, we used GWAS metadata, tissue samples from pathologically confirmed frontotemporal lobar degeneration, and *in silico* techniques to identify and characterize protein interaction networks associated with svPPA risk. We identified 64 svPPA risk genes that interact at the protein level. The protein pathways represented in this svPPA gene network are critical regulators of RNA metabolism and cell death, such as SMAD proteins and NOTCH1. Many of the genes in this network are involved in TDP-43 metabolism. Contrary to the conventional notion that svPPA is a clinical syndrome with few genetic risk factors, our analyses show that svPPA risk is complex and polygenic in nature. Risk for svPPA is likely driven by multiple common variants in genes interacting with TDP-43, along with cell death,x` working in combination to promote neurodegeneration.

## Introduction

Frontotemporal lobar dementia (FTLD) is a heterogeneous family of progressive neurodegenerative disorders characterized by degeneration of the frontal and temporal lobes with corresponding clinical deficits in social processes, language, and executive functioning^1^. One of the most common FTLD syndromes, semantic variant of primary progressive aphasia (svPPA; also referred to as semantic dementia (SD)) preferentially affects language and semantic processing^2,3^. svPPA is unique amongst the FTLD spectrum disorders because the vast majority of cases have TAR DNA-binding protein 43 (TDP-43) positive inclusions, with a very small fraction showing other protein pathologies^2,4^. TDP-43 is a protein heavily involved in RNA metabolic processes including transcription, splicing, and transport^5^. Despite its relatively consistent clinical presentation and predictable pathological features, little is known about the genetic factors underlying risk for svPPA^6^.

svPPA poses a unique problem and opportunity amongst the FTLD spectrum disorders^2^. When contrasted to pathologically diverse syndromes within the FTLD spectrum such as behavioral variant frontotemporal dementia (bvFTD), the relative clinical and pathological homogeneity of svPPA (typically TDP-43 Type C) could suggest a shared genetic risk profile across patients. However, very few, if any, common variants have been shown to contribute to the sporadic form of svPPA^6,7^. This observation is striking when compared to other forms of FTLD in which up to 40% of patients have a positive family history and there are many known, common genetic risk variants^1,7,8^. This conundrum suggests, among other possibilities, that svPPA risk is more strongly influenced by environmental or developmental factors such as handedness^9^ and/or that svPPA is by nature highly polygenic. Identifying the genetic contributions to disease is critical as it provides insight into the causal biology underlying deterministic neurodegenerative pathways.

Recent advances have enabled the analysis of multiple sub-GWAS significant loci by integrating heterogeneous risk alleles with experimentally validated outside reference data^10^. This not only increases statistical power, but also overcomes challenges such as locus heterogeneity to determine novel loci underlying disease risk. We have previously utilized these methods to successfully identify new risk genes and inform the pathobiology of complex diseases like multiple sclerosis^11^. This approach is particularly powerful because it relies upon previously validated experimental data to link disease-associated genes with one another, further corroborating the biological relevance of risk loci. In this study, we focused our analyses on svPPA not only because it is pathologically homogeneous, but also because previous efforts to identify genetic risk factors associated with svPPA have been limited by small sample sizes amongst single risk loci. Utilizing polygenic strategies to identify risk factors for svPPA presents a unique opportunity, as knowledge gleaned from these analyses could also inform other forms of FTLD resulting from TDP-43 pathology.

## Results

This study utilized summary statistics of the phase-1 GWAS data from the International FTD-Genomics Consortium (IFGC), comprised of 2,154 clinically diagnosed FTD spectrum cases and 4,308 controls and a total of 6,026,384 SNPs. Of the 2,154 cases diagnosed with FTD, 361 were diagnosed with the svPPA subtype (referred to as “semantic dementia” in the original study). Cases within the cohort were diagnosed according to the Neary criteria for FTLD^12^. For additional cohort details, please see Ferrari *et al*.^6^.

**Figure 1:**
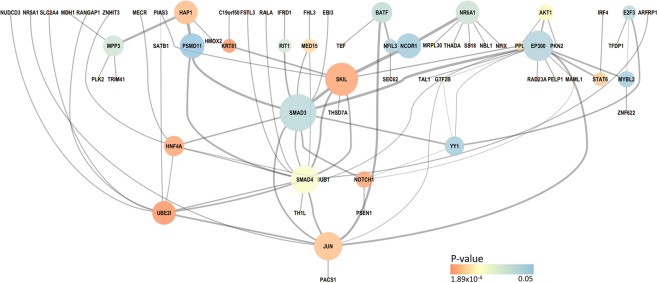
svPPA Network. Network results are shown for protein interaction network based pathway analysis (PINBPA) in the semantic variant of primary progressive aphasia compared to controls. The network genes are color coded according to their respective p-value (see Methods), with warmer colors indicating p-values closer to the minimum value of 1.89E-4 and cooler colors indicating p-values closer to the maximum p-value of 0.05. The size of a node corresponds to closeness centrality (a metric that describes a node’s nearness to other network nodes). The thickness of edges in the network corresponds to edge betweenness (a metric that describes the number of paths going through an edge in the network).

We generated gene-based p-values for 17,466 genes with data available in the svPPA cohort using the tool versatile gene-based association study (VEGAS). We next generated protein interaction networks (PINs) for the significant genes (VEGAS p < 0.05) using the protein interaction network-based pathway analysis (PINBPA) package. The background PIN database used in our analyses contained 8,960 proteins and 27,724 interactions. The largest network generated (in terms of both nodes and edges) contained 64 nodes (genes) and had 81 edges (protein interactions) (Fig. 1, Supplementary Table S1). We evaluated only the largest and most significant network to avoid false positive findings. Notably, *TARDBP* (the gene encoding TDP-43) was absent from our network, but many genes implicated in cell death (e.g. *SMAD3*, *SMAD4*), nuclear trafficking (e.g. *RANGAP1*), and stress responses (e.g. *HNF4A*) were present. The svPPA network was within the top 10^th^ percentile for both nodes and edges based on permutation testing.

We next explored the gene expression patterns of the svPPA network genes in pathologically confirmed cohorts of FTLD cases versus controls. Fifty-eight of 64 svPPA genes in the dataset (GSE13162) had expression data available. Fifteen of the svPPA network genes (*HNF4A, NR5A1, TAL1, SLC2A4, PSEN1, KRT81, MYBL2, UBE2I, EBI3, BATF, ARFRP1, NR6A1, PACS1, PELP1*, and *TEF*) were significantly differentially expressed at an FDR-corrected p < 0.05 in cases when compared to controls (Table 1, Supplementary Table S2). For each gene in the svPPA network we provide a detailed summary of our results with respect to VEGAS results, the top 3 regions expressing each gene in healthy human brain tissue (from the Braineac cohort, http://braineac.org), OMIM biological process implicated for each gene, and known neurodegenerative disease associations in Supplementary Table S3.

**Table 1:** Differential Expression Analyses in FTLD-U Cases.

Gene Name	Raw P-value	FDR Corrected P-value	SE	Beta
*HNF4A*	2.16E-05	1.25E-03	0.195	−1.53
*NR5A*	1.84E-04	3.55E-03	0.199	−1.31
*TAL1*	9.96E-05	2.89E-03	0.287	−1.37
*SLC2A4*	7.36E-04	0.01	0.237	−1.16
*PSEN1*	2.12E-03	0.02	0.605	1.04
*KRT81*	3.63E-03	0.03	0.293	−0.981
*MYBL2*	3.13E-03	0.03	0.251	−0.999
*UBE2I*	3.51E-03	0.03	0.284	−0.985
*EBI3*	4.64E-03	0.03	0.375	−0.952
*BATF*	6.06E-03	0.03	0.256	−0.920
*ARFRP1*	6.57E-03	0.03	0.240	−0.910
*NR6A1*	0.01	0.04	0.218	−0.860
*PACS1*	0.01	0.04	0.192	−0.855
*PELP1*	0.01	0.04	0.246	−0.8.71
*TEF*	0.01	0.05	0.109	−0.835

Expression analyses revealed 15 out of 64 genes in our svPPA network showed dysregulated expression in pathologically confirmed cases of frontotemporal lobar degeneration with ubiquitinated inclusions (FTLD-U) when compared to age-matched controls. P-values for the top associated probe for each gene (FDR corrected p < 0.05 to account for multiple testing) are shown. FDR – false discovery rate.

To better understand the biological and functional implications of the svPPA gene network, we performed two separate ontological analyses. The first analysis utilized two common and publicly available databases of gene ontologies (Reactome and Gene Ontology [GO] portals)^13,14^. For the second analysis, we used a recently developed and independent analytical pipeline called weighted protein-protein interaction network analysis (W-PPI-NA) pipeline, which was recently developed by our group^15,16^.

Sixty-four genes were included in the first svPPA ontological pathway analysis (Supplementary Table S1); genetic enrichment was seen in pathways involved in RNA metabolism, development, immunity, and cell signaling (Table 2 and Supplementary Data S1). Reactome pathways highlighted in our enrichment analysis included broad classifications at the nucleotide level such as nucleotide excision repair, as well as more specific processes including SMAD signaling proteins (fold enrichment = 56.11, p = 1.65 × 10^−3^), NOTCH (fold enrichment = 66.75, p = 9.56 × 10^−7^), and Activin (fold enrichment = 74.46, p = 1.79 × 10^−2^) (summarized in Table 2, full results in Supplementary Table S4).

**Table 2:** Reactome Pathway Analysis: Genes Implicated in svPPA Protein Network.

Biological Pathway	Pathway	# genes in ref, candidate dataset	Candidate Genes Mapped	Fold Enriched	P-value
**RNA Transcription**					
	SMAD transcriptional activity	23, 4	*SMAD3, SMAD4, NCOR1, SKIL*	56.11	1.65 × 10^−3^
	AP-2 (TF-AP2) transcription regulation	34, 4	*EP300, UBE2I, YYI, MYBL2*	37.96	7.67 × 10^−3^
	NOTCH1 transcription	45,4	*EP300, NCOR1, NOTCH1, MAML1*	28.6	2.29 × 10^−2^
	Nucleotide excision repair	110, 5	*EP300, UBE2I, YYI, RAD23A, PIAS3*	14.67	4.48 × 10^−2^
**Cell signaling**					
	Pre-NOTCH transcription and translation	29, 6	*EP300, TDFP1, NOTCH1, JUN, E2F3, MAML1*	66.75	9.56 × 10^−7^
	Diseases of signal transduction	282, 8	*EP300, SMAD3, NCOR1, SMAD4, NOTCH1, PSMD11, AKT1, MAML1*	9.15	4.84 × 10^−3^
	Activin beta signaling pathway	13, 3	*SMAD3, SMAD4, FSTL3*	74.46	1.79 × 10^−2^
**Development/Growth**					
	Developmental biology	11676, 21	*EP300, SMAD3, YYI, NCOR1, SLC2A4, SMAD4, NOTCH1, MED15, PSMD11, JUN, AKT1, NR5A1*	4.80	1.11 × 10^−2^

Pathway analysis results are shown. For each broad biological pathway, specific pathways from Reactome databases are shown. In all analyses, the p-value presented has been adjusted using the Bonferroni technique. Please see Supplementary Table S3 for additional details.

**Figure 2:**
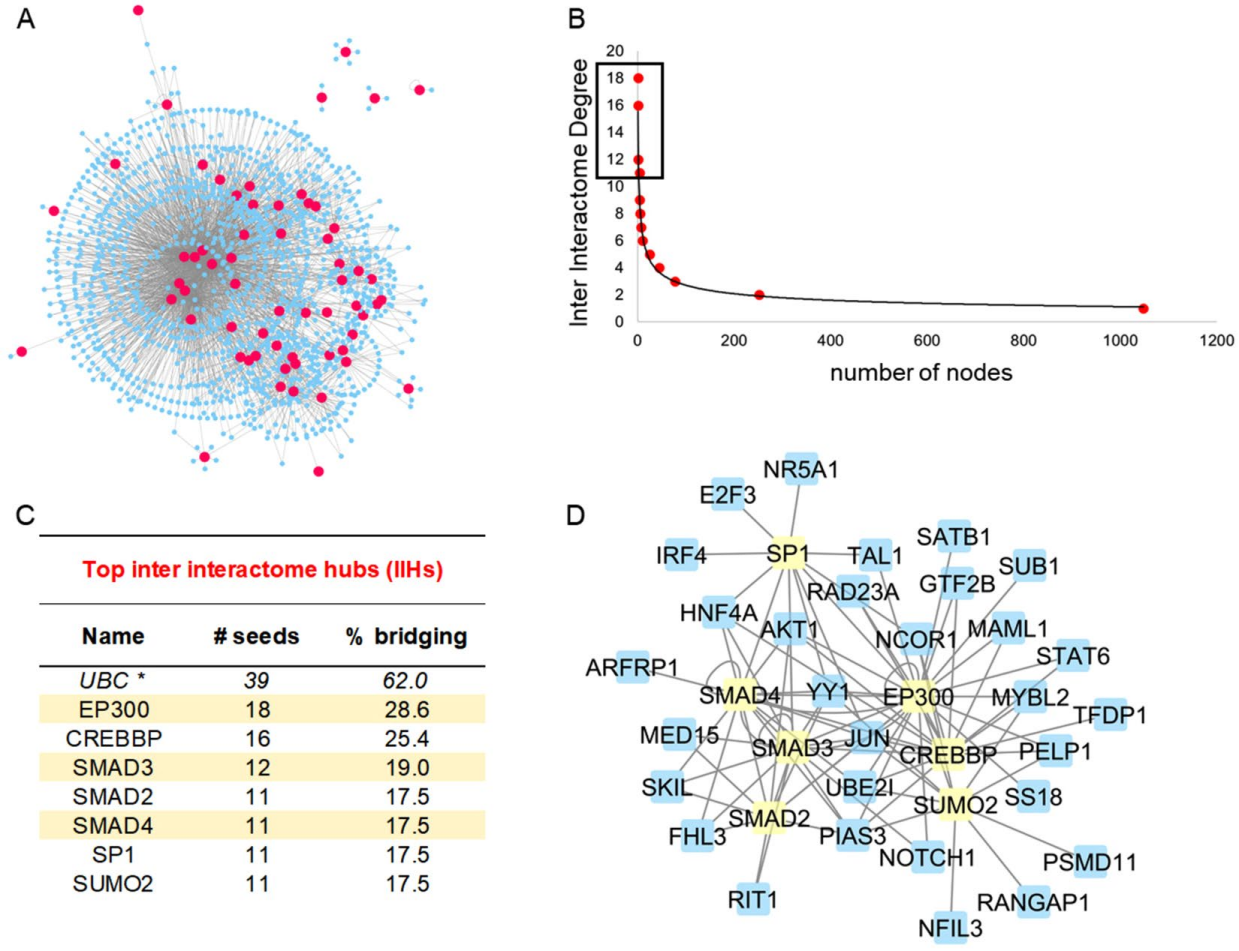
svPPA Interactome Analyses. (**A**) Results from the weighted protein-protein interaction network analysis (W-PPI-NA) pipeline are shown for the 64 genes identified using protein interaction network based pathway analysis (PINBPA). Seeds in the results are shown in pink while interactors are shown in blue. (**B**) The inter-interactome degree distribution curve illustrates the quantity of nodes on the x-axis that bridged to the quantity of seeds (shown on the y-axis). The inter-interactome hubs (IIHs) are marked by a rectangle. (**C**) The IIHs with their associated number of bridged seeds and percent bridging are shown. *The protein UBC is reported but ignored given that it could indicate nonspecific ubiquitin binding to unrelated proteins marked for degradation (see^15^ for further information). (**D**) The network core (depicted in yellow) around the IIHs.

**Figure 3:**
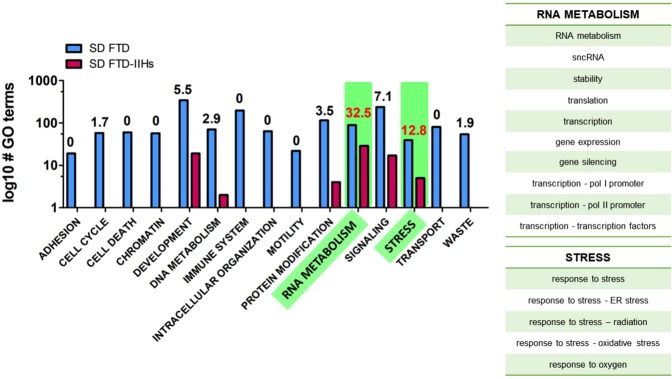
g:profiler Biological Pathway Analyses. Comparison of the g:Profiler functional enrichment performed for the entire weighted protein-protein interaction network analysis (W-PPI-NA) network (blue bars) and the core network (red bars). Gene ontology (GO) terms are reported on the y-axis and functional blocks are reported on the x-axis. The number on top of the bars indicates the percentage of overlap for each single functional block; the numbers in red indicate more than 12% of conservation (significant conservation). Each significant functional block is made of the semantic classes reported below the graph.

Our second svPPA ontological pathway analysis – through the W-PPI-NA pipeline – enabled the independent topological characterization of our 64 svPPA network genes. Provided that one svPPA network risk gene – *TH1L* – did not survive after applying the W-PPI-NA method, the (second) network was ultimately generated using 63 proteins as seeds. We merged the annotations reported in multiple protein-protein interaction (PPI) databases within the IMEX consortium^17^. After filtering and scoring the protein network, the interactome was composed of 1,495 nodes and 2,407 edges where all but 4 nodes (FSTL3, MRPL30, NR6A1 and EBI3) were interconnected (Fig. 2A and Supplementary Fig. S1). Since one protein in the network, UBC, tags protein targets for degradation, it might non-specifically bind any protein in the sub-cellular environment and not necessarily represent a specific functional pathway. We thus excluded UBC from the network’s statistics. We identified the inter-interactome hubs (IIHs) (n = 7) as the core of the network with the highest interconnectivity (Fig. 2D); these nodes were able to bridge over 15% of the entire interactomes (Fig. 2B,C). By comparing the core of the network with randomly sampled parts of the network, we verified that the IIHs-driven network was indeed the most densely connected (Supplementary Fig. S2). The core of the network was made of 37 nodes (7 IIHs and their interactors) and 93 edges. These were strongly interconnected (average number of neighbors = 4.7). We next functionally annotated the interactomes, focusing on GO-BPs (biological processes) using g:Profiler. The first functional enrichment aforementioned in this paragraph was followed by a second iteration of the same procedure but only applied on the densely connected core of the network (Fig. 2D). Our results (Supplementary Data S1) indicated a list of semantic classes that were a subset of the former. Interestingly the subset terms (percentage of retention >12%, i.e. an arbitrary yet robust threshold that takes into account the functions containing the largest number of replicated BP terms in our experimental setting (Fig. 3)) pointed to the following functional blocks: i) ‘RNA metabolism’ and ii) ‘stress’ (Fig. 3) as the common functions characterizing that part of the protein network with strongest cohesion among the initial seeds. Of note, key players within these functional blocks were members of the SMAD protein family. We were thus able to replicate the results obtained through the Reactome analyses using a completely independent and different approach, further supporting the biological roles of svPPA network risk genes.

## Discussion

Our analyses revealed a polygenic network of 64 svPPA risk genes which interact at the protein level. Many of these genes are differentially expressed in pathologically confirmed cases of FTLD with ubiquitin-positive inclusions (the same pathology most commonly seen in svPPA). Finally, we examined the biological pathways seen in this network and found significant enrichment in processing and metabolism of RNA as well as cell stress and apoptosis. These findings show that svPPA risk variants cluster in biological pathways representing processes closely tied to the primary protein pathology (TDP-43) seen in svPPA. Furthermore, our results suggest that further study of common genetic variation in svPPA could prove useful in the identification of individuals at risk for disease.

Our ontological pathway analysis showed the greatest degree of enrichment in pathways related to transcription and RNA metabolism. Converging evidence from multiple studies supports the role of RNA dysmetabolism in the pathogenesis of svPPA^2,18–20^. The most common protein pathology seen in svPPA is TDP-43 Type C^2^. TDP-43 is a protein heavily involved in RNA metabolic processes including transcription, splicing, and transport^5^. Thus, our finding of ontology enrichment in pathways related to RNA metabolism may be particularly relevant to svPPA which, in contrast to many other FTLD-spectrum disorders, is associated with a relatively low frequency of pathological accumulations of tau^4^. Recent work has shown that the RNA ribonuclear protein hnRNP E2 is associated specifically with TDP-43 immunoreactive neurites in svPPA, but not with other pathological FTLD subtypes^21^. Interestingly, 11 of our 64 genes for svPPA have been previously reported to have statistically significant co-expression profiles associated with *hnRNP E2* (*AKT1, GTF2B, MAML1, MDH1, RAD23A, RANGAP1, SMAD3, STAT6, TFDP1, UBE21, ZNHIT3*)^21^. A number of these genes have been previously implicated in other TDP-43 proteinopathies without an svPPA syndrome. For example, in *Drosophila* RANGAP1 is a suppressor of neurotoxicity due to *C9ORF72* pathogenic hexanucleotide repeat expansion^22^. Lastly, many of the genes in our svPPA network have been previously shown to be targets of neuronal TDP-43 ribonucleoprotein complexes, including *AKT1, NOTCH1*, and *PSEN1*^23^.

Other biological pathways enriched in our analysis provided further support for a TDP proteinopathy-mediated mechanism of disease. For example, we observed enrichment in *SMAD* signaling pathways. SMAD proteins regulate the expression of target genes critical for regulating neuronal stability and apoptosis^24^. Previous work in mouse models has shown that TDP-43 aggregates co-localize with phosphorylated Smad proteins, which mediate downstream signaling in the transforming growth factor beta (TGF-beta) pathway^25^. TGF-beta acts through the TGF-beta type II receptor that forms a complex with Activin, another pathway highly enriched in our Reactome pathway analysis. This pathway plays a role in a number of biological functions including neuronal development and homeostasis^26,27^. Furthermore, activation of TGF-beta and SMAD signaling has been shown to reduce mislocalized TDP-43 aggregate formation in human cell culture^28^. Lastly, Notch signaling pathways, including the gene *NOTCH1*, a key molecule regulating neuronal health and homeostasis, were significantly overrepresented in our svPPA risk gene network. Notch dysregulation has been previously reported as a mechanism of neurodegeneration seen in cases with *PSEN1* mutations resulting in an FTD-like syndrome, though the pathology associated with these mutations is unreported^29^.

Our study benefits from its use of multiple publicly available, well-validated cohorts. The network analysis techniques used in this study rely upon previously validated experimental protein interaction data, which means the network interactions shown are ripe targets for cellular and molecular studies. This study is limited by a lack of additional GWAS data in which to replicate our genetic findings, but the anticipated release of IFGC phase III data will provide a suitable cohort for future confirmation and elaboration of these findings. The protein interaction analyses in our study rely heavily upon preexisting data and could therefore bias our findings towards the most studied biological pathways and processes. Additional studies focused on alternative ontological categories such as cellular components and molecular function may prove informative in future studies, but in our study the results of these ontological categories were judged as too general to be informative (data not shown). Unfortunately, the results of our VEGAS analyses do not facilitate the calculation of each gene’s, or the overall network’s, percent contribution to svPPA risk. Further molecular and model-organism studies will be required to validate and prove the importance of our svPPA network genes as modifiers of disease risk in svPPA and other TDP-43 proteinopathies. We attempted to replicate the protein interaction network results from our VEGAS analysis using differential expression data from GSE13162. The network generated using differential expression from GSE13162 was not significant after multiple testing correction (data not shown). This study focused on one of the FTLD phenotypes. Ongoing work in our group focuses on the other IFGC phenotypes^30^ as well as the work of other groups which will help to elucidate the genetic and biochemical pathways that make svPPA distinct from the other FTLD phenotypes and may further highlight which processes are shared across FTLD subtypes.

In summary, this study identified and bioinformatically characterized a network of 64 svPPA risk genes with interacting protein products. Many of these genes were differentially expressed in pathologically confirmed FTLD cases. Common variation in svPPA risk genes is implicated in RNA metabolism and cell death signaling. These findings are an important step towards a genetic understanding of what was previously considered a disease largely due to environmental and other risk factors.

## Methods

### Ethics, consent and permissions

This study was performed in accordance with the guidelines set forth by the University of California, San Francisco Human Research Protection Program Institutional Review Board. The data collection from the original GWAS used in this analysis was overseen by the relevant institutional review boards, and ethics committees approved the research protocol of all individual studies used in the current analysis. Participants of those studies provided written informed consent.

### svPPA gene network generation

To generate the svPPA network, we first calculated gene-level significance values using VEGAS^10^. This tool uses location information from the UCSC Genome Browser (hg18) assembly to assign individual SNPs to their respective gene. Gene boundaries were defined as 50 kb beyond the 5′ and 3′ UTRs of each gene to ensure we captured the effects of regulatory regions and SNPs in linkage disequilibrium (LD). VEGAS accounts for background LD patterns between markers within a gene using data from individuals of northern and western European descent (HapMap2 CEU)^31^. Monte-Carlo simulations use these LD patterns to generate a multivariate normal distribution which is used to calculate an empirical p-value for each gene. For additional details on VEGAS, please see Liu *et al*. and Baranzini *et al*.^10,32^.

We derived PINs from the iRefIndex database, a collection of 15 human PIN data sets from different sources^33^. The combined dataset from these PINs contained over 400,000 interactions among approximately 25,000 proteins. To minimize the number of false positives in our PIN, we limited our PIN to interactions described in at least two independent publications. The resulting network used as a background network in our analyses contained 8,960 proteins and 27,724 interactions. The PIN was uploaded into Cytoscape^34^ version 2.8.2 and used PINBPA to label each entry with genomic position, gene p-value, association block membership, and gene name (node attribute).

We computed significant first-order interactions by filtering the main network so that only the genes (and their protein products) with a VEGAS p-value less than 0.05 were retained. Following this, the number of resulting nodes and edges along with the size of the largest connected component were computed in Cytoscape (http://www.cytoscape.org/). We evaluated the network strength using permutations. The p-values from our VEGAS analyses were mixed randomly amongst genes and permuted networks to create a null distribution. The results of our svPPA network were compared against this null distribution. We evaluated the largest and most significant network to avoid false positive findings.

### Gene expression in pathologically confirmed FTLD cases

We hypothesized that genes from our network analysis would be dysregulated in pathologically confirmed cases of FTLD as compared to controls. Given that svPPA is primarily associated with ubiquitinated inclusions composed of TDP-43, we chose to use a publicly available dataset of individuals diagnosed with FTLD with ubiquitinated inclusions and comparable control cases. Ten of the participants were pathologically diagnosed with sporadic FTLD and 11 diagnosed as controls (Gene Expression Omnibus (GEO) dataset GSE13162)^35^. In linear regression models we controlled for sex, post mortem interval, and age.

### svPPA biological pathway enrichment analysis

We performed enrichment analyses on our genes of interest using the Reactome and GO annotation databases. Reactome (v60, released April 20^th^, 2017) is a curated pathway database that aggregates human pathways and reactions from UniProt, Ensembl, KEGG, GO, and PubMed, among others (http://www.reactome.org). We restricted the analysis to comparisons within the *Homo sapiens* annotations and ran the statistical enrichment tests for biological pathways under the default settings (which corrects for multiple testing using the Bonferroni technique).

To replicate the findings of our primary ontological analysis, we next applied the recently developed W-PPI-NA pipeline^15^ to increase resolution on the candidate proteins isolated by PINBPA. Specifically, we generated a second independent network using the svPPA genes prioritized by the PINBPA analysis by extracting (12 May 2017) their protein interactors (PPIs) from the following databases within the IMEX consortium^17^: APID Interactomes, BioGrid, bhf-ucl, InnateDB, InnateDB-All, IntAct, mentha, MINT, InnateDB-IMEx, UniProt, and MBInfo by means of the “PSICQUIC” R package (version 1.15.0 by Paul Shannon, http://code.google.com/p/psicquic/). PPIs were harmonized by converting Protein IDs to UniProt and Entrez IDs thus allowing merging of all databases. We removed TrEMBL, non-protein interactors (e.g. chemicals), obsolete Entrez, and Entrez matching to multiple Swiss-Prot identifiers. All PPIs underwent quality control and filtering leading to removal of: i) all the non-human taxid annotations, and ii) all the annotations with multiple or no PubMed identifiers or no description of Interaction Detection Method. The interactions were then scored as follows: (i) evaluation of the number of different publications reporting the interaction and (ii) evaluation of the number of different methods reporting the interaction. All the interactors with a final score ≤2 were discarded to reduce false positive rate. The final network was visualized using Cytoscape and analyzed through the network analysis plug-in. The inter-interactome degree distribution curve was drawn considering all the nodes within the network and the number of seeds they connect (number of node edges/number of network seeds = connection degree). We defined IIHs as any node connecting more than 15% of the seed’s interactomes.

As part of the W-PPI-NA, we applied functional annotation analysis to the network built using the PINBPA-prioritized genes as seeds. We then performed Gene Ontology (GO) biological processes (BPs) enrichment analyses through g:Profiler (g:GOSt,http://biit.cs.ut.ee/gprofiler/)^36^, which runs Fisher’s one-tailed test and uses a set counts and sizes (SCS) based technique for multiple test correction. The statistical domain size was only annotated genes; no hierarchical filtering was included. We then grouped enriched GO-BP terms into custom-made “semantic classes” (Supplementary Data S2). We removed general, thus negligible, semantic classes such as general, metabolism, enzymes, protein modification, and physiology. Semantic classes were further grouped by similarity in more general classes called functional blocks.

## Supplementary information

Supplement

## Data Availability

Requests for GWAS metadata should be directed to the International FTD-Genomics Consortium. The PINBPA package is available through Cytoscape (www.cytoscape.org). Reactome data is available at www.reactome.org. PANTHER data is available at www.pantherdb.org. The “PSICQUIC” R package (version 1.15.0 by Paul Shannon) is available at (http://code.google.com/p/psicquic/). g:Profiler is available at(g:GOSt,http://biit.cs.ut.ee/gprofiler/).
