# Supplementary material for: Genetic variation across RNA metabolism and cell death gene networks is implicated in the semantic variant of primary progressive aphasia
Source: Sci Rep. 2019 Jul 26;9:10854. doi: 10.1038/s41598-019-46415-1 (PMC6659677; doi:10.1038/s41598-019-46415-1)
Supplement: Supplementary file 1 — Supplement [file 41598_2019_46415_MOESM1_ESM.pdf]

Supplemental Information:

Genetic variation across RNA metabolism and cell death gene networks is implicated in the semantic variant of primary progressive aphasia

Luke W Bonham[1,2†], Natasha ZR Steele[1†], Celeste M Karch[3], Iris Broce[2], Ethan G Geier[1], Natalie L Wen[3], Parastoo Momeni[4], John Hardy[5], International FTD-Genomics Consortium (IFGC)°, Zachary A Miller[1], Marilu Gorno-Tempini[1], Christopher P Hess[2], Patrick Lewis[5,6], Bruce L Miller[1], William W Seeley[1], Claudia Manzoni[5,6], Rahul S Desikan[2], Sergio E Baranzini[7], Raffaele Ferrari[5], Jennifer S Yokoyama[1]

[†]These authors contributed equally to the manuscript

SUPPLEMENTAL TABLES

Supplementary Table S1. Full PINBPA Results

Gene Name	Chromosome	Description	Ensembl ID	HGNC ID	HPRD ID	MIM ID	Gene P-value	snpP-value
HAP1	17	huntingtin-associated protein 1	ENSG00000173805	4812	2972	600947	6.13E-03	3.55E-03
ZNHIT3	17	"zinc finger, HIT type 3"	ENSG00000108278	12309	5139	604500	1.11E-03	9.79E-05
NFIX	19	nuclear factor I/X (CCAAT-binding transcription factor)	ENSG00000008441	7788	1234	164005	9.94E-04	1.74E-03
SKIL	3	SKI-like oncogene	ENSG00000136603	10897	1319	165340	2.65E-03	8.90E-04
TRIM41	5	tripartite motif-containing 41	ENSG00000146063	19013	15558	610530	5.40E-04	9.65E-03
SMAD4	18	SMAD family member 4	ENSG00000141646	6770	2995	600993	0.02	0.01
SMAD3	15	SMAD family member 3	ENSG00000166949	6769	4380	603109	0.04	1.06E-04
NBL1	1	"neuroblastoma, suppression of tumorigenicity 1"	ENSG00000158747	7650	9001	600613	0.03	5.30E-03
PACS1	11	phosphofurin acidic cluster sorting protein 1	ENSG00000175115	30032	6320	607492	0.01	1.75E-03
MYBL2	20	v-myb myeloblastosis viral oncogene homolog (avian)-like 2	ENSG00000101057	7548	3247	601415	0.04	0.02
E2F3	6	E2F transcription factor 3	ENSG00000112242	3115	2693	600427	0.04	8.32E-04
MED15	22	mediator complex subunit 15	ENSG00000099917	14248	12117	607372	0.01	2.45E-03
EBI3	19	Epstein-Barr virus induced 3	ENSG00000105246	3129	12051	605816	0.02	8.82E-03
SUB1	5	SUB1 homolog (S. cerevisiae)	ENSG00000113387	19985	2737	600503	0.03	4.64E-03
PELP1	17	"proline, glutamate and leucine rich protein 1"	ENSG00000141456	30134	10144	609455	0.01	4.38E-03
MECR	1	mitochondrial trans-2-enoyl-CoA reductase	ENSG00000116353	19691	12192	608205	0.02	1.61E-03
FHL3	1	four and a half LIM domains 3	ENSG00000183386	3704	9101	602790	0.04	2.52E-03
RIT1	1	Ras-like without CAAX 1	ENSG00000143622	10023	10195	609591	0.03	8.67E-03
NR6A1	9	"nuclear receptor subfamily 6, group A, member 1"	ENSG00000148200	7985	4147	602778	0.03	9.15E-03
ARFRP1	20	ADP-ribosylation factor related protein 1	ENSG00000101246	662	5264	604699	1.19E-03	1.83E-04

TFDP1	13	transcription factor Dp-1	ENSG00000198176	11749	1792	189902	9.54E-03	1.22E-04
THSD7A	7	"thrombospondin, type I, domain containing 7A"	ENSG00000005108	22207		612249	0.03	8.04E-05
UBE2I	16	"ubiquitin-conjugating enzyme E2I (UBC9 homolog, yeast)"	ENSG00000103275	12485	9045	601661	1.89E-04	5.23E-04
AKT1	14	v-akt murine thymoma viral oncogene homolog 1	ENSG00000142208	391	1261	164730	0.01	0.02
NCOR1	17	nuclear receptor co-repressor 1	ENSG00000141027	7672	2911	600849	0.04	0.04
GTF2B	1	general transcription factor IIB	ENSG00000137947	4648	8928	189963	0.01	8.76E-03
PSEN1	14	presenilin 1	ENSG00000080815	9508	87	104311	0.03	6.29E-03
BATF	14	"basic leucine zipper transcription factor, ATF-like"	ENSG00000156127	958	9820	612476	0.04	1.16E-03
PPL	16	periplakin	ENSG00000118898	9273	4187	602871	0.01	1.45E-04
HNF4A	20	"hepatocyte nuclear factor 4, alpha"	ENSG00000101076	5024	2612	600281	2.48E-03	3.96E-04
SLC2A4	17	"solute carrier family 2 (facilitated glucose transporter), member 4"	ENSG00000181856	11009	688	138190	1.60E-03	2.64E-03
FSTL3	19	follistatin-like 3 (secreted glycoprotein)	ENSG00000070404	3973	5632	605343	5.45E-04	3.60E-04
EP300	22	E1A binding protein p300	ENSG00000100393	3373	4078	602700	0.04	6.22E-03
MRPL30	2	mitochondrial ribosomal protein L30	ENSG00000185414	14036	14751	611838	0.02	0.02
RAD23A	19	RAD23 homolog A (S. cerevisiae)	ENSG00000179262	9812	7191	600061	9.94E-04	2.67E-03
TAL1	1	T-cell acute lymphocytic leukemia 1	ENSG00000162367	11556	1753	187040	0.02	0.01
ZNF622	5	zinc finger protein 622	ENSG00000173545	30958	12278	608694	5.47E-03	9.54E-04
HMOX2	16	heme oxygenase (decycling) 2	ENSG00000103415	5014	783	141251	7.45E-03	2.42E-03
C19orf50	19		ENSG00000105700	28420	14526		0.01	6.44E-03
NFIL3	9	"nuclear factor, interleukin 3 regulated"	ENSG00000165030	7787	5619	605327	0.04	0.01
KRT81	12	keratin 81	ENSG00000205426	6458	3691	602153	3.16E-04	2.07E-05
PLK2	5	polo-like kinase 2 (Drosophila)	ENSG00000145632	19699	6118	607023	0.01	3.97E-03

PKN2	1	protein kinase N2	ENSG00000065243	9406	3972	602549	0.01	5.05E-03
RALA	7	v-ral simian leukemia viral oncogene homolog A (ras related)	ENSG00000006451	9839	1549	179550	4.59E-04	3.49E-05
PSMD11	17	"proteasome (prosome, macropain) 26S subunit, non-ATPase, 11"	ENSG00000108671	9556	5119	604449	0.05	5.69E-04
TH1L	20	TH1-like (Drosophila)	ENSG00000101158	15934	16096	605297	0.01	1.45E-03
NUDCD3	7	NudC domain containing 3	ENSG00000015676	22208	13831	610296	0.03	8.04E-03
IFRD1	7	interferon-related developmental regulator 1	ENSG00000006652	5456	4612	603502	5.91E-03	2.19E-04
STAT6	12	"signal transducer and activator of transcription 6, interleukin-4 induced"	ENSG00000166888	11368	3302	601512	7.84E-03	7.55E-03
RANGAP1	22	Ran GTPase activating protein 1	ENSG00000100401	9854	3839	602362	8.51E-03	8.37E-03
SEC62	3	SEC62 homolog (S. cerevisiae)	ENSG00000008952	11846	3706	602173	2.65E-03	1.50E-03
SATB1	3	SATB homeobox 1	ENSG00000182568	10541	3647	602075	0.02	3.86E-04
YY1	14	YY1 transcription factor	ENSG00000100811	12856	2482	600013	0.04	0.03
MAML1	5	mastermind-like 1 (Drosophila)	ENSG00000161021	13632	5665	605424	8.61E-03	2.30E-03
TEF	22	thyrotrophic embryonic factor	ENSG00000167074	11722	1783	188595	8.51E-03	1.33E-03
MPP3	17	"membrane protein, palmitoylated 3 (MAGUK p55 subfamily member 3)"	ENSG00000161647	7221	3069	601114	0.03	0.01
JUN	1	jun oncogene	ENSG00000177606	6204	1302	165160	6.46E-03	4.57E-03
SS18	18	"synovial sarcoma translocation, chromosome 18"	ENSG00000141380	11340	2559	600192	1.82E-03	1.63E-03
MDH1	2	"malate dehydrogenase 1, NAD (soluble)"	ENSG00000014641	6970	1100	154200	1.09E-03	2.64E-04
NOTCH1	9	"Notch homolog 1, translocation-associated (Drosophila)"	ENSG00000148400	7881	1827	190198	2.65E-03	3.28E-03
NR5A1	9	"nuclear receptor subfamily 5, group A, member 1"	ENSG00000136931	7983	1702	184757	0.03	9.15E-03
PIAS3	1	"protein inhibitor of activated STAT, 3"	ENSG00000131788	16861	9068	605987	0.04	0.03

IRF4	6	interferon regulatory factor 4	ENSG00000137265	6119	3543	601900	5.40E-04	7.75E-05
THADA	2	thyroid adenoma associated	ENSG00000115970	19217	18177	611800	0.01	3.25E-04

Supplemental Table S1 Legend. Canonical name of gene SNPs, Band – chromosomal locationlocation, Block # – block association membership defined as groups of sequential genes with a p-value of <.05, Chrom – chromosomal location, ENSMBL ID – annotation ID in ENSMBL, HGNC ID – Human genome nomenclature ID, HPRD ID – Human protein reference database, MIMID – Mendelian inheritance in man ID, P-value – Gene p value, Top SNP p-value – Top p value for SNP

Supplementary Table S2. Full Expression Results in Pathologically Confirmed FTLD-U Cases

Gene Symbol	Beta	SE	P-Value	P-Value (FDR)
AKT1	-0.021	0.26	0.948	0.948
ARFRP1	-0.91	0.24	0.00657	0.0346
BATF	-0.92	0.256	0.00606	0.0346
E2F3	-0.492	0.53	0.128	0.226
EBI3	-0.952	0.375	0.00464	0.0299
EP300	-0.624	0.561	0.0561	0.136
FHL3	-0.398	0.257	0.217	0.331
FSTL3	-0.59	0.305	0.0703	0.157
GTF2B	0.675	0.393	0.0397	0.11
HAP1	-0.799	0.299	0.0159	0.0577
HMOX2	0.449	0.541	0.165	0.273
HNF4A	-1.53	0.195	0.0000216	0.00125
IFRD1	0.0831	0.733	0.795	0.863
IRF4	-0.664	0.0901	0.0428	0.113
JUN	0.0433	0.857	0.892	0.924
KRT81	-0.981	0.293	0.00363	0.0263
MAML1	-0.314	0.624	0.328	0.449
MDH1	0.365	0.75	0.256	0.363
MECR	0.0992	0.487	0.756	0.843
MED15	-0.376	0.198	0.242	0.36
MPP3	-0.265	1.12	0.408	0.504
MYBL2	-0.999	0.251	0.00313	0.0263
NBL1	0.0268	0.537	0.933	0.948
NCOR1	0.566	0.35	0.082	0.168
NFIL3	0.122	1.1	0.701	0.814
NOTCH1	0.0444	0.407	0.889	0.924
NR5A1	-1.31	0.199	0.000184	0.00355
NR6A1	-0.86	0.218	0.00989	0.0426
NUDCD3	0.115	0.443	0.718	0.816
PACS1	-0.855	0.192	0.0103	0.0426
PELP1	-0.871	0.246	0.00905	0.0426
PIAS3	0.147	0.356	0.646	0.765
PKN2	-0.723	0.69	0.0282	0.0962
PLK2	0.279	2.26	0.384	0.494
PPL	0.566	0.797	0.0819	0.168
PSEN1	1.04	0.605	0.00212	0.0246
PSMD11	0.296	0.281	0.356	0.469

RAD23A	0.371	0.374	0.249	0.361
RALA	0.626	0.316	0.0555	0.136
RANGAP1	-0.41	0.381	0.204	0.319
RIT1	0.465	0.451	0.15	0.256
SATB1	-0.511	0.763	0.115	0.208
SEC62	0.562	0.407	0.0841	0.168
SKIL	-0.69	0.0925	0.0357	0.11
SLC2A4	-1.16	0.237	0.000736	0.0107
SMAD3	0.59	0.292	0.0702	0.157
SMAD4	0.555	0.661	0.0881	0.17
SS18	0.674	0.59	0.0399	0.11
STAT6	-0.258	0.446	0.42	0.508
SUB1	0.685	0.521	0.0368	0.11
TAL1	-1.37	0.287	0.0000996	0.00289
TEF	-0.835	0.109	0.012	0.0465
TFDP1	-0.0795	0.389	0.803	0.863
THADA	0.522	0.239	0.108	0.202
THSD7A	0.433	0.62	0.179	0.289
UBE2I	-0.985	0.284	0.00351	0.0263
YY1	0.311	0.327	0.333	0.449
ZNHIT3	0.265	0.484	0.408	0.504

Supplementary Table S2 Legend. Expression analyses revealed 22 out of 64 genes in our SD network showed nominally significant dysregulated expression in pathologically confirmed cases of frontotemporal dementia with ubiquinitated inclusions (FTLD-U) vs age-matched controls.

Supplemental Figure 3: Gene level side by side comparison of 64 VEGAS identified genes with OMIM biological processes and previous implications in degenerative disease

Gene Name	Chr. #	Description	Ensembl ID	HGNC ID	HPRD ID	MIM ID	Top Snp	Gene P-value	Snp P-value	BraineactID	Start Location	Stop Location	Number of Probe Sets	Region Rank 1	Region Rank 2	Region Rank 3	OMIM Biological Process	Degenerative Disease/Process Association
AKT1	14	v-akt murine thymoma viral oncogene homolog 1	ENSG00000142208	391	1261	164730	rs12432802	0.01	0.02	3581132	10523695	105262055	33	OCTX	FCTX	HIPP	Growth factor-induced neuronal survival	Alzheimer Disease,[1] Frontotemporal dementia,[2] Huntington Disease,[3] Parkinson Disease[4]
ARFRP1	20	ADP-ribosylation factor related protein 1	ENSG00000101246	662	5264	604699	rs11696871	<0.01	<0.01	3914072	62330007	62339365	18	WHMT	OCTX	TCTX	Lipolysis	None reported
BATF	14	"basic leucine zipper transcription factor, ATF-like"	ENSG00000156127	958	9820	612476	rs175722	0.04	<0.01	3544605	75940385	76013335	7	PUTM	WHMT	FCTX	Hematopoetic stem cell transcription factor, Telomere length	None reported
C19orf50	19		ENSG00000105700	28420	14526	na	rs10854166	0.01	0.01	3825141	18668598	18680176	12	WHMT	MEDU	SNIG	Lysosomal function	Retinal Degeneration[5]
E2F3	6	E2F transcription factor 3	ENSG00000112242	3115	2693	600427	rs746720	0.04	<0.01	2897576	20393801	20493940	11	HIPP	FCTX	PUTM	Cell cycle	Neuroinflammation[6]
EBI3	19	Epstein-Barr virus induced 3	ENSG00000105246	3129	12051	605816	rs7253162	0.02	0.01	3817380	4229299	4244057	5	SNIG	WHMT	THAL	Immune regulation	Alzheimer Disease[7]
EP300	22	E1A binding protein p300	ENSG00000100393	3373	4078	602700	rs5758211	0.04	0.01	3946615	41487790	41576079	44	CRBL	WHMT	OCTX	Cell proliferation and differentiation	None reported
FHL3	1	four and a half LIM domains 3	ENSG00000183386	3704	9101	602790	rs4440824	0.04	<0.01	2407478	38462445	38471310	6	MEDU	SNIG	WHMT	Immune regulation	None reported
FSTL3	19	follistatin-like 3 (secreted glycoprotein)	ENSG00000070404	3973	5632	605343	rs13730	<0.01	<0.01	3815097	674029	683385	8	WHMT	MEDU	HIPP	Cell proliferation and differentiation	None reported
GTF2B	1	general transcription factor IIB	ENSG00000137947	4648	8928	189963	rs11809597	0.01	0.01	2421753	89318321	89357607	9	FCTX	TCTX	OCTX	Transcription initiation and regulation	Huntington Disease,[8] Spinocerebellar ataxia, Machado-Joseph Disease[9]
HAP1	17	huntingtin-associated protein 1	ENSG00000173805	4812	2972	600947	rs4796691	0.01	<0.01	3757288	39873994	39890926	16	SNIG	HIPP	MEDU	Cellular trafficking	Alzheimer Disease[10]
HMOX2	16	heme oxygenase (decycling) 2	ENSG00000103415	5014	783	141251	rs1684600	0.01	<0.01	3678186	4558594	4588778	13	OCTX	THAL	FCTX	Heme degradation	None reported
HNF4A	20	hepatocyte nuclear factor 4, alpha	ENSG00000101076	5024	2612	600281	rs11699901	<0.01	<0.01	3886453	42984100	43061484	23	FCTX	SNIG	HIPP	Hepatic metabolism	Duchenne Muscular Dystrophy[11]
IFRD1	7	interferon-related developmental regulator 1	ENSG00000006652	5456	4612	603502	rs2520482	0.01	<0.01	3019519	112048229	112116552	19	WHMT	HIPP	SNIG	Cell proliferation and differentiation	Neuroinflammation[12]
IRF4	6	interferon regulatory factor 4	ENSG00000137265	6119	3543	601900	rs6900384	<0.01	<0.01	2891341	384725	411640	16	WHMT	MEDU	THAL	Immune regulation	ALS [13]

JUN	1	jun oncogene	ENSG00000177606	6204	1302	165160	rs2760496	0.01	<0.01	2415084	59246386	59268803	14	HIPP	FCTX	TCTX	Cell cycle	None reported
KRT81	12	keratin 81	ENSG00000205426	6458	3691	602153	rs3741722	<0.01	<0.01	3455261	52679699	52702146	6	FCTX	HIPP	TCTX	Keratin production	Neurodegeneration[14]
MAML1	5	mastermind-like 1 (Drosophila)	ENSG00000161021	13632	5665	605424	rs12374446	0.01	<0.01	2844410	179159851	179219189	12	CRBL	WHMT	OCTX	Cell cycle	Alzheimer Disease,[15] Parkinson Disease,[15] ALS[15]
MDH1	2	"malate dehydrogenase 1, NAD (soluble)"	ENSG00000014641	6970	1100	154200	rs1869700	<0.01	<0.01	2485176	63772665	63835619	14	OCTX	TCTX	FCTX	Cell proliferation and differentiation	Child-onset dystonia with optic atrophy and basal ganglia degeneration[16]
MECR	1	mitochondrial trans-2-enoyl-CoA reductase	ENSG00000116353	19691	12192	608205	rs2068388	0.02	<0.01	2403793	29518582	29557444	16	FCTX	HIPP	TCTX	Mitochondrial metabolism	None reported
MED15	22	mediator complex subunit 15	ENSG00000099917	14248	12117	607372	rs12484992	0.01	<0.01	3937587	20847344	20941902	29	TCTX	FCTX	OCTX	Gene transcription	Neurodegeneration[17]
MPP3	17	"membrane protein, palmitoylated 3 (MAGUK p55 subfamily member 3)"	ENSG00000161647	7221	3069	601114	rs8081646	0.03	0.01	3758640	41878177	41910537	23	CRBL	MEDU	HIPP	Cell membrane function	Alzheimer Disease,[18] Parkinson Disease[19]
MRPL30	2	mitochondrial ribosomal protein L30	ENSG00000185414	14036	14751	611838	rs2632277	0.02	0.02	2495758	99755892	99768503	10	WHMT	CRBL	HIPP	Mitochondrial metabolism	Alzheimer Disease[20]
MYBL2	20	v-myb myeloblastosis viral oncogene homolog (avian)-like 2	ENSG00000101057	7548	3247	601415	rs619289	0.04	0.02	3886223	42294384	42345118	17	WHMT	THAL	SNIG	Cell cycle	None reported
NBL1	1	"neuroblastoma, suppression of tumorigenicity 1"	ENSG00000158747	7650	9001	600613	rs2854108	0.03	0.01	2323774	19751280	19985905	9	FCTX	TCTX	OCTX	Cell cycle	Huntington Disease[21]
NCOR1	17	nuclear receptor co-repressor 1	ENSG00000141027	7672	2911	600849	rs3785631	0.04	0.04	3746881	15542407	16121039	52	CRBL	OCTX	TCTX	Gene transcription	ALS,[22] Neurodegeneration[22]
NFIL3	9	"nuclear factor, interleukin 3 regulated"	ENSG00000165030	7787	5619	605327	rs1591069	0.04	0.01	3214451	94171343	94187333	5	CRBL	OCTX	THAL	Immune regulation	Cerebellar degeneration[23]
NFIX	19	nuclear factor I/X (CCAAT-binding transcription factor)	ENSG00000008441	7788	1234	164005	rs8103241	<0.01	<0.01	3822122	13106604	13209605	23	WHMT	OCTX	CRBL	Gene transcription	Alzheimer Disease[24]
NOTCH1	9	"Notch homolog 1, translocation-associated (Drosophila)"	ENSG00000148400	7881	1827	190198	rs10116408	<0.01	<0.01	3230141	139388898	139440238	35	WHMT	MEDU	SNIG	Cytoskeletal function	Alzheimer Disease,[25] Prion disease, Neurodegeneration
NR5A1	9	"nuclear receptor subfamily 5, group A, member 1"	ENSG00000136931	7983	1702	184757	rs7851142	0.03	0.01	3225058	127243515	127269699	11	SNIG	WHMT	PUTM	Gene transcription	Neurodegeneration[26]
NR6A1	9	"nuclear receptor subfamily 6, group A, member 1"	ENSG00000148200	7985	4147	602778	rs7851142	0.03	0.01	3225096	127279564	127534695	16	CRBL	TCTX	OCTX	Gene transcription, neurogenesis	Neurodegeneration[27]
NUDCD3	7	NudC domain containing 3	ENSG00000015676	22208	13831	610296	rs10280989	0.03	0.01	3048631	44421883	44530397	21	OCTX	FCTX	TCTX	Cytoskeletal function	Alzheimer Disease[28]
PACS1	11	phosphofurin acidic cluster sorting protein 1	ENSG00000175115	30032	6320	607492	rs2452681	0.01	<0.01	3335952	65837748	66012438	26	PUTM	HIPP	FCTX	Cellular trafficking	Neurodegeneration[29]

PELP1	17	"proline, glutamate and leucine rich protein 1"	ENSG00000141456	30134	10144	609455	rs4522461	0.01	<0.01	3742236	4574688	4607712	19	WHMT	FCTX	OCTX	Cell cycle and gene expression	None reported
PIAS3	1	"protein inhibitor of activated STAT, 3"	ENSG00000131788	16861	9068	605987	rs11577342	0.04	0.03	2432647	145585835	145621863	32	CRBL	PUTM	FCTX	Immune regulation	Alzheimer Disease,[30] ALS[30]
PKN2	1	protein kinase N2	ENSG00000065243	9406	3972	602549	rs10489889	0.01	0.01	2345617	89101172	89301918	29	WHMT	CRBL	SNIG	Neuronal cytoskeletal organization	Parkinson Disease,[31] Alzheimer Disease[32]
PLK2	5	polo-like kinase 2 (Drosophila)	ENSG00000145632	19699	6118	607023	rs10071783	0.01	<0.01	2858023	57749815	57756867	15	PUTM	HIPP	TCTX	Cell division	None reported
PPL	16	periplakin	ENSG00000118898	9273	4187	602871	rs2075639	0.01	<0.01	3678462	4931712	4987140	23	OCTX	TCTX	FCTX	Keratin production	Alzheimer Disease,[33] Frontotemporal dementia[34]
PSEN1	14	presenilin 1	ENSG00000080815	9508	87	104311	rs362398	0.03	0.01	3543481	73602818	73690398	23	WHMT	MEDU	SNIG	Chromosome organization and segregation	Frontotemporal dementia,[35] Alzheimer Disease[35]
PSMD11	17	"proteasome (prosome, macropain) 26S subunit, non-ATPase, 11"	ENSG00000108671	9556	5119	604449	rs4794922	0.05	<0.01	3717737	30770974	30810336	17	TCTX	HIPP	FCTX	Proteasome function	Neurodegeneration,[36] ALS[36]
RAD23A	19	RAD23 homolog A (S. cerevisiae)	ENSG00000179262	9812	7191	600061	rs1548546	<0.01	<0.01	3822074	13056654	13064768	11	FCTX	OCTX	TCTX	Proteasome function, DNA repair	None reported
RALA	7	v-ral simian leukemia viral oncogene homolog A (ras related)	ENSG00000006451	9839	1549	179550	rs17171603	<0.01	<0.01	2998404	39662526	39821761	12	MEDU	WHMT	SNIG	Mitochondrial metabolism	ALS,[37] Frontotemporal dementia[38]
RANGAP1	22	Ran GTPase activating protein 1	ENSG00000100401	9854	3839	602362	rs1953	0.01	0.01	3961842	41639496	41698081	22	OCTX	TCTX	FCTX	Cell signaling	Hippocampal degeneration[39]
RIT1	1	Ras-like without CAAX 1	ENSG00000143622	10023	10195	609591	rs822503	0.03	0.01	2437736	155869377	155881167	8	SNIG	MEDU	PUTM	Cell survival	ALS, Alzheimer Disease[40]
SATB1	3	SATB homeobox 1	ENSG00000182568	10541	3647	602075	rs17519462	0.02	<0.01	2665199	18389431	18487082	23	OCTX	FCTX	TCTX	Gene expression	None reported
SEC62	3	SEC62 homolog (S. cerevisiae)	ENSG00000008952	11846	3706	602173	rs2072576	<0.01	<0.01	2651782	169683752	169711959	14	CRBL	WHMT	TCTX	Gene expression	None reported
SKIL	3	SKI-like oncogene	ENSG00000136603	10897	1319	165340	rs4955588	<0.01	<0.01	2651989	170075513	170111436	13	TCTX	FCTX	SNIG	Cell proliferation and differentiation	Alzheimer Disease[41]
SLC2A4	17	"solute carrier family 2 (facilitated glucose transporter), member 4"	ENSG00000181856	11009	688	138190	rs222852	<0.01	<0.01	3708399	7184977	7191556	15	SNIG	PUTM	THAL	Cell proliferation and differentiation	Frontotemporal dementia[42]
SMAD3	15	SMAD family member 3	ENSG00000166949	6769	4380	603109	rs16950615	0.04	<0.01	3598959	67357989	67509764	20	PUTM	FCTX	TCTX	Cell proliferation and differentiation	Frontotemporal dementia[42]
SMAD4	18	SMAD family member 4	ENSG00000141646	6770	2995	600993	rs2202980	0.02	0.01	3788302	48554802	48607297	19	CRBL	HIPP	PUTM	Cell proliferation and differentiation	None reported
SS18	18	"synovial sarcoma translocation, chromosome 18"	ENSG00000141380	11340	2559	600192	rs4800708	<0.01	<0.01	3802129	23450259	23671399	19	WHMT	MEDU	SNIG	Transcription initiation and regulation	Alzheimer Disease[43]

STAT6	12	"signal transducer and activator of transcription 6, interleukin-4 induced"	ENSG00000166888	11368	3302	601512	rs17119386	0.01	0.01	3458337	57488868	57516709	25	FCTX	TCTX	PUTM	Immune regulation	None reported
SUB1	5	SUB1 homolog (S. cerevisiae)	ENSG00000113387	19985	2737	600503	rs648658	0.03	<0.01	2805581	32522864	32627015	5	FCTX	TCTX	HIPP	Transcription initiation and regulation	None reported
TAL1	1	T-cell acute lymphocytic leukemia 1	ENSG00000162367	11556	1753	187040	rs2070929	0.02	0.01	2411198	47681966	47698294	14	SNIG	THAL	WHMT	Immune regulation	None reported
TEF	22	thyrotrophic embryonic factor	ENSG00000167074	11722	1783	188595	rs2073167	0.01	<0.01	3946817	41763482	41795325	15	CRBL	PUTM	OCTX	Transcription initiation and regulation	None reported
TFDP1	13	transcription factor Dp-1	ENSG00000198176	11749	1792	189902	rs12020712	0.01	<0.01	3502710	114238807	114302563	12	CRBL	WHMT	MEDU	Cell cycle	None reported
TH1L	20	TH1-like (Drosophila)	ENSG00000101158	15934	16096	605297	rs4812048	0.01	<0.01	3891278	57555821	57571779	26	WHMT	CRBL	MEDU	Transcription initiation and regulation	None reported
THADA	2	thyroid adenoma associated	ENSG00000115970	19217	18177	611800	rs7589033	0.01	<0.01	2550542	43457634	43823177	46	WHMT	PUTM	SNIG	Apoptosis	None reported
THSD7A	7	"thrombospondin, type I, domain containing 7A"	ENSG00000005108	22207		612249	rs83	0.03	<0.01	3038664	11251133	11901972	31	THAL	OCTX	TCTX	Cell migration	Prion disease[44]
TRIM41	5	tripartite motif-containing 41	ENSG00000146063	19013	15558	610530	rs254460	<0.01	0.01	2845043	180649519	180662790	18	WHMT	MEDU	THAL	Protein modification	None reported
UBE2I	16	"ubiquitin-conjugating enzyme E2I (UBC9 homolog, yeast)"	ENSG00000103275	12485	9045	601661	rs6600128	<0.01	<0.01	3643703	1349450	1377015	19	CRBL	HIPP	FCTX	Cell signaling	Alzheimer Disease[7]
YY1	14	YY1 transcription factor	ENSG00000100811	12856	2482	600013	rs3825555	0.04	0.03	3551677	100680630	100749115	14	FCTX	TCTX	OCTX	Immune regulation	None reported
ZNF622	5	zinc finger protein 622	ENSG00000173545	30958	12278	608694	rs4702146	0.01	<0.01	2849960	16409108	16466047	8	SNIG	TCTX	THAL	Transcription initiation and regulation	Cerebellar degeneration[45]

Supplemental Table S3 Legend. Additional results summarizing and annotating the VEGAS and PINBPA results are shown. For each gene, we provide the full name and alternate identifiers along with VEGAS summary statistics, the top 3 regions expressing each gene in healthy human tissue, OMIM biological process implicated for each gene, and known neurodegenerative disease associations are shown. In order to analyze the location and expression patterns of the svPPA network in healthy human tissue, we used the Braineac Database (www.braineac.org) from the UK Brain Expression Consortium (UKBEC). The dataset contains mRNA quantification data from 134 brains from neuropathologically confirmed healthy individuals from the MRC Sudden Death Brain Bank in Edinburgh, UK, and the Sun Health Research Institute in Sun City West, USA. Ten

regions of the brain were available for analysis: occipital cortex, frontal cortex, temporal cortex, hippocampus, intralobular white matter, cerebellar cortex, thalamus, putamen, substantia nigra, and medulla. RNA expression quantification was assessed using the Affymetrix Exon 1.0 ST Array following the manufacturer's protocols. For full details on RNA extraction, QC processes, and information regarding analysis of array data see (www.braineac.org). Braineac combines probe sets to create a gene expression profile for each gene using a Winsorized mean across the available probe-sets. We retrieved the expression data for each of the 64 genes in the svPPA network. When multiple expression profiles were available for a given gene, we chose the profile with the largest number of probes for inclusion in our analyses. Chr # – chromosomal number, ENSMBL ID – annotation ID in ENSMBL, HGNC ID – Human genome nomenclature ID, HPRD ID – Human protein reference database, MIMID – Mendelian inheritance in man ID, P-value – Gene p value, Top SNP p-value – Top p value for SNP, Braineac ID – Braineac reference database, Start location– Start of chromosomal sequence location, Stop location– End of chromosomal sequence location, Region ranks 1-3 – Top three brain regions where gene is expressed, CB – Cerebellar Cortex, FL – Frontal lobe cortex, HIP – Hippocampus, MED – Medulla, OL – Occipital lobe cortex, PUT – Putamen, SN – Substantia Nigra, TL – Temporal lobe cortex, OMIM Biological Process –Online Mendelian Inheritance in Man biological process reported for each gene.

REFERENCES

1. Rickle, A. *et al.* Akt activity in Alzheimer's disease and other neurodegenerative disorders. *Neuroreport* **15,** 955–959 (2004).
2. Ferrari, R. *et al.* Frontotemporal dementia: Insights into the biological underpinnings of disease through gene co-expression network analysis. *Mol. Neurodegener.* **11,** (2016).
3. Colin, E. *et al.* Akt is altered in an animal model of Huntington's disease and in patients. *Eur. J. Neurosci.* **21,** 1478–1488 (2005).
4. Greene, L. A., Levy, O. & Malagelada, C. Akt as a victim, villain and potential hero in Parkinson's disease pathophysiology and treatment. *Cell. Mol. Neurobiol.* **31,** 969–978 (2011).
5. Chen, D., Chen, Y., Forrest, D. & Bremner, R. E2f2 induces cone photoreceptor apoptosis independent of E2f1 and E2f3. *Cell Death Differ.* **20,** 931–940 (2013).
6. Tirotta, E. *et al.* Epstein-Barr virus-induced gene 3 negatively regulates neuroinflammation and T cell activation following coronavirus-induced encephalomyelitis. *J. Neuroimmunol.* **254,** 110–116 (2013).
7. Aubry, S. *et al.* Assembly and interrogation of Alzheimer's disease genetic networks reveal novel regulators of progression. *PLoS One* **10,** (2015).
8. Li, S. *et al.* Lack of Huntingtin-Associated Protein-1 Causes Neuronal Death Resembling Hypothalamic Degeneration in Huntington ' s Disease. *J. Neurosci.* **23,** 6956–6964 (2003).
9. Prigge, J. R. & Schmidt, E. E. HAP1 can sequester a subset of TBP in cytoplasmic inclusions via specific interaction with the conserved TBPCORE. *BMC Mol. Biol.* **8,** (2007).
10. Atamna, H. & Frey, W. H. A role for heme in Alzheimer's disease: Heme binds amyloid   and has altered metabolism. *Proc. Natl. Acad. Sci.* **101,** 11153–11158 (2004).

11. Nelson, S. F. & Griggs, R. C. Predicting the severity of duchenne muscular dystrophy: Implications for treatment. *Neurology* **76,** 208–209 (2011).
12. Carriba, P. & Comella, J. X. Neurodegeneration and neuroinflammation: Two processes, one target. *Neural Regeneration Research* **10,** 1581–1583 (2015).
13. Schenkel, J. Activation of the c-Jun transcription factor following neurodegeneration in vivo. *Neurosci. Lett.* **361,** 36–39 (2004).
14. Just Ribeiro, M. Saint, Hansson, M. L., Lindberg, M. J., Popko-Ścibor, A. E. & Wallberg, A. E. GSK3β is a negative regulator of the transcriptional coactivator MAML1. *Nucleic Acids Res.* **37,** 6691–6700 (2009).
15. Recabarren, D. & Alarcón, M. Gene networks in neurodegenerative disorders. *Life Sciences* **183,** 83–97 (2017).
16. Heimer, G. *et al.* MECR Mutations Cause Childhood-Onset Dystonia and Optic Atrophy, a Mitochondrial Fatty Acid Synthesis Disorder. *Am. J. Hum. Genet.* **99,** 1229–1244 (2016).
17. Haberman, R. P. *et al.* Prominent hippocampal CA3 gene expression profile in neurocognitive aging. *Neurobiol. Aging* **32,** 1678–1692 (2011).
18. Lunnon, K. *et al.* Mitochondrial genes are altered in blood early in Alzheimer's disease. *Neurobiol. Aging* **53,** 36–47 (2017).
19. Pinho, R. *et al.* Gene expression differences in peripheral blood of Parkinson's disease patients with distinct progression profiles. *PLoS One* **11,** (2016).
20. Han, Z., Huang, H., Gao, Y. & Huang, Q. Functional annotation of Alzheimer's disease associated loci revealed by GWASs. *PLoS One* **12,** (2017).
21. Yohrling IV, G. J., Farrell, L. A., Hollenberg, A. N. & Cha, J. H. J. Mutant huntingtin increases nuclear corepressor function and enhances ligand-dependent nuclear hormone receptor activation. *Mol. Cell. Neurosci.* **23,** 28–38 (2003).
22. Tamai, S. I. *et al.* Neuroprotective role of the basic leucine zipper transcription factor NFIL3 in models of amyotrophic lateral sclerosis. *J. Biol. Chem.* **289,** 1629–1638 (2014).
23. Becherel, O. J. *et al.* A new model to study neurodegeneration in ataxia oculomotor apraxia type 2. *Hum. Mol. Genet.* **24,** 5759–5774 (2015).
24. Caberlotto, L., Marchetti, L., Lauria, M., Scotti, M. & Parolo, S. Integration of transcriptomic and genomic data suggests candidate mechanisms for APOE4-mediated pathogenic action in Alzheimer's disease. *Sci. Rep.* **6,** 32583 (2016).
25. Ambegaokar, S. S. & Jackson, G. R. Functional genomic screen and network analysis reveal novel modifiers of tauopathy dissociated from tau phosphorylation. *Hum. Mol. Genet.* **20,** 4947–4977 (2011).
26. Olivares, A. M., Moreno-Ramos, O. A. & Haider, N. B. Role of nuclear receptors in central nervous system development and associated diseases. *Journal of Experimental Neuroscience* **9s2,** 93–121 (2015).

27. Taipale, M. *et al.* A quantitative chaperone interaction network reveals the architecture of cellular protein homeostasis pathways. *Cell* **158,** 434–448 (2014).
28. Wan, L. *et al.* PACS-1 defines a novel gene family of cytosolic sorting proteins required for trans-Golgi network localization. *Cell* **94,** 205–216 (1998).
29. Monteiro, M. C., Coleman, M. D., Hill, E. J., Prediger, R. D. & Maia, C. S. F. Neuroprotection in Neurodegenerative Disease: From Basic Science to Clinical Applications. *Oxidative Medicine and Cellular Longevity* **2017,** (2017).
30. Thauerer, B., Nedden, S. & Baier-bitterlich, G. Protein Kinase C-Related Kinase (PKN/PRK). Potential Key-Role for PKN1 in Protection of Hypoxic Neurons. *Curr. Neuropharmacol.* **12,** 213–218 (2014).
31. Inglis, K. J. *et al.* Polo-like kinase 2 (PLK2) phosphorylates α-synuclein at serine 129 in central nervous system. *J. Biol. Chem.* **284,** 2598–2602 (2009).
32. Looyenga, B. D. & Brundin, P. Silencing synuclein at the synapse with PLK2. *Proc. Natl. Acad. Sci.* **110,** 16293–16294 (2013).
33. Kelleher, R. J. & Shen, J. Presenilin-1 mutations and Alzheimer's disease. *Proc. Natl. Acad. Sci.* **114,** 629–631 (2017).
34. Watanabe, H., Xia, D., Kanekiyo, T., Kelleher, R. J. & Shen, J. Familial Frontotemporal Dementia-Associated Presenilin-1 c.548G>T Mutation Causes Decreased mRNA Expression and Reduced Presenilin Function in Knock-In Mice. *J. Neurosci.* **32,** 5085–5096 (2012).
35. Lokireddy, S., Kukushkin, N. V. & Goldberg, A. L. cAMP-induced phosphorylation of 26S proteasomes on Rpn6/PSMD11 enhances their activity and the degradation of misfolded proteins. *Proc. Natl. Acad. Sci.* **112,** E7176–E7185 (2015).
36. Jablonski, A. M. *et al.* Loss of RAD-23 Protects Against Models of Motor Neuron Disease by Enhancing Mutant Protein Clearance. *J. Neurosci.* **35,** 14286–14306 (2015).
37. Mihevc, S. P. *et al.* Nuclear trafficking in amyotrophic lateral sclerosis and frontotemporal lobar degeneration. *Brain* **140,** 13–26 (2017).
38. Gao, F., Almeida, S. & Lopez-Gonzalez, R. Dysregulated molecular pathways in amyotrophic lateral sclerosis–frontotemporal dementia spectrum disorder. *EMBO J.* e201797568 (2017). doi:10.15252/embj.201797568
39. Mir, S., Cai, W. & Andres, D. A. RIT1 GTPase regulates Sox2 transcriptional activity and hippocampal neurogenesis. *J. Biol. Chem.* **292,** 2054–2064 (2017).
40. Hokama, M. *et al.* Altered expression of diabetes-related genes in Alzheimer's disease brains: The Hisayama study. *Cereb. Cortex* **24,** 2476–2488 (2014).
41. Nelson, A. R. *et al.* Neurovascular dysfunction and neurodegeneration in dementia and Alzheimer's disease. *BBA - Mol. Basis Dis.* **1862,** 887–900 (2016).
42. Bonham, L. W. *et al.* Protein network analysis reveals selectively vulnerable regions and biological processes in

FTD. *Neurol. Genet.* **4,** e266 (2018).
43. Bhattarai, P. *et al.* IL4/STAT6 Signaling Activates Neural Stem Cell Proliferation and Neurogenesis upon Amyloid-β42 Aggregation in Adult Zebrafish Brain. *Cell Rep.* **17,** 941–948 (2016).
44. Booth, S. *et al.* Identification of central nervous system genes involved in the host response to the scrapie agent during preclinical and clinical infection. *J. Gen. Virol.* **85,** 3459–3471 (2004).
45. Anttonen, A. K. *et al.* ZNHIT3 is defective in PEHO syndrome, a severe encephalopathy with cerebellar granule neuron loss. *Brain* **140,** 1267–1279 (2017).

Supplementary Table S4: Reactome gene ontology pathways for semantic dementia

Reactome pathways	Reference list	# Genes overlapping	# Genes expected	Fold Enrichment	P-value
Signaling by Activin (R-HSA-1502540)	13	3	0.04	74.46	1.79E-02
Pre-NOTCH Transcription and Translation (R-HSA-1912408)	29	6	0.09	66.75	9.56E-07
Downregulation of SMAD2/3:SMAD4 transcriptional activity (R-HSA-2173795)	23	4	0.07	56.11	1.65E-03
Pre-NOTCH Expression and Processing (R-HSA-1912422)	45	6	0.14	43.02	1.28E-05
Transcriptional regulation by the AP-2 (TFAP2) family of transcription factors (R-HSA-8864260)	34	4	0.11	37.96	7.67E-03
Transcriptional activity of SMAD2/SMAD3:SMAD4 heterotrimer (R-HSA-2173793)	44	5	0.14	36.66	5.37E-04
NOTCH1 Intracellular Domain Regulates Transcription (R-HSA-2122947)	45	4	0.14	28.68	2.29E-02
Signaling by TGF-beta Receptor Complex (R-HSA-170834)	72	5	0.22	22.41	5.89E-03
Signaling by NOTCH (R-HSA-157118)	108	7	0.33	20.91	9.14E-05
Nucleotide Excision Repair (R-HSA-5696398)	110	5	0.34	14.67	4.48E-02
Diseases of signal transduction (R-HSA-5663202)	282	8	0.87	9.15	4.84E-03
Generic Transcription Pathway (R-HSA-212436)	834	17	2.58	6.58	8.26E-07
Developmental Biology (R-HSA-1266738)	806	12	2.5	4.8	1.11E-02
Disease (R-HSA-1643685)	899	12	2.79	4.31	3.29E-02
Gene Expression (R-HSA-74160)	1693	21	5.25	4	3.58E-05
Unclassified (UNCLASSIFIED)	11676	21	36.19	0.58	0.00E+00

Supplementary Table S4 Legend. Reactome pathways highlighted in our ontological analysis and corresponding pathway reference numbers. Reference list – number of genes documented

in the Reactome database for each pathway, # Genes overlapping – number of genes in both pathway reference list and set of enriched svPPA genes, # Genes expected – Reactome a priori expected degree of overlap given number of genes entered into the pathway analysis tool, Fold enrichment – Reactome degree of enrichment based on expected value and number of overlapping genes, p value – Bonferroni corrected p values.

SUPPLEMENTAL FIGURES

Supplementary Figure 1. Interactome Results

A

Top 10 interactomes by dimension	
**Seed**	**# direct interactors**
EP300	311
JUN	234
AKT1	181
UBE2I	174
SMAD3	169
SMAD4	135
NCOR1	100
HNF4A	68
YY1	65
PSMD11	53

B

Contribution to the network	
**# Seeds**	**percentage contribution**
1	>10 %
1	>8 %
3	>7 %
2	>4 %
3	>2 %
17	>1 %
11	>0.5 %
25	>0.1 %

C

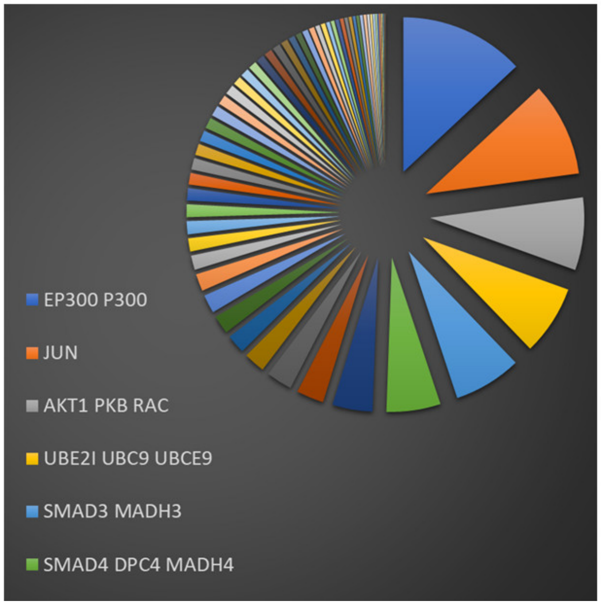

Supplemental Figure 1 Legend. Detailed interactome analysis results are shown. (A) The top 10 interactomes ranked by number of interactors. (B) Percentage contribution to the network for each seed in the analysis is shown. (C) Graphical depiction of the results shown in (A).

Supplemental Figure 2. Comparison of svPPA network against randomly sampled networks

Dataset	Number of seeds	Subnetwork nodes around seeds	Number of edges	Average number of neighbors	Network density
**random 1**	7	14	8	1.143	0.088
**random 2**	7	29	30	1.793	0.064
**random 3**	7	17	11	1.294	0.081
**random 4**	7	47	46	1.957	0.043
**random 5**	7	16	11	1.375	0.092
**random 6**	70	145	172	2.193	0.015
**random 7**	70	133	146	2.165	0.016
**random 8**	70	142	150	2.014	0.014
**random 9**	70	195	223	2.133	0.011
**random 10**	70	149	146	1.933	0.013
**SD-FTD IIHs**	7	37	93	4.703	0.131

Supplemental Figure 2 Legend. Results for the comparison of svPPA (SD-FTD) network against randomly sampled parts of the network.

IFGC APPENDICES

APPENDIX A

Acknowledgments

Intramural funding from the National Institute of Neurological Disorders and Stroke (NINDS) and National Institute on Aging (NIA), the Wellcome/MRC Centre on Parkinson's disease, Alzheimer's Research UK (ARUK, Grant ARUK-PG2012-18) and by the office of the Dean of the School of Medicine, Department of Internal Medicine, at Texas Tech University Health Sciences Center.

We thank Mike Hubank and Kerra Pearce at the Genomic core facility at the Institute of Child Health (ICH), University College of London (UCL), for assisting RF in performing Illumina genotyping experiments (FTD-GWAS genotyping). This study utilized the high-performance computational capabilities of the Biowulf Linux cluster at the National Institutes of Health, Bethesda, Md. (http://biowulf.nih.gov). North American Brain Expression Consortium (NABEC) - The work performed by the North American Brain Expression Consortium (NABEC) was supported in part by the Intramural Research Program of the National Institute on Aging, National Institutes of Health, part of the US Department of Health and Human Services; project number ZIA AG000932-04. In addition this work was supported by a Research Grant from the Department of Defense, W81XWH-09-2-0128. UK Brain Expression Consortium (UKBEC) - This work performed by the UK Brain Expression Consortium (UKBEC) was supported by the MRC through the MRC Sudden Death Brain Bank (C.S.), by a Project Grant (G0901254 to J.H. and M.W.) and by a Fellowship award (G0802462 to M.R.). D.T. was supported by the King Faisal Specialist Hospital and Research Centre, Saudi Arabia. Computing facilities used at King's College London were supported by the National Institute for Health Research (NIHR) Biomedical Research Centre based at Guy's and St Thomas' NHS Foundation Trust and King's College London. We would like to thank AROS Applied Biotechnology AS company laboratories and Affymetrix for their valuable input. RF's work is supported by Alzheimer's Society (grant number 284), UK; JBJK was supported by the National Health and Medical Resarch Council (NHMRC) Australia, Project Grants 510217 and 1005769; CDS was supported by NHMRC Project Grants 630428 and 1005769; PRS was supported by NHMRC Project Grants 510217 and 1005769 and acknowledges that DNA samples were prepared by Genetic Repositories Australia, supported by NHMRC Enabling Grant 401184; GMH was supported by NHMRC Research Fellowship 630434, Project Grant 1029538, Program Grant 1037746; JRH was supported by the Australian Research Council Federation Fellowship, NHMRC Project Grant 1029538, NHMRC Program Grant 1037746; OP was supported by NHMRC Career Development Fellowship 1022684, Project Grant 1003139. IH, AR and MB acknowledge the patients and controls who participated in this project and the Trinitat Port-Carbó and her family who are supporting Fundació ACE research programs. CC was supported by Grant P30-NS069329-01 and acknowledges that the recruitment and clinical characterization of research participants at Washington University were supported by NIH P50 AG05681, P01 AG03991, and P01 AG026276. LB and GB were supported by the Ricerca Corrente, Italian Ministry of Health; RG was supported by Fondazione CARIPLO 2009-2633, Ricerca Corrente, Italian

Ministry of Health; GF was supported by Fondazione CARIPLO 2009-2633. ES was supported by the Italian Ministry of Health; CF was supported by Fondazione Cariplo; MS was supported from the Italian Ministry of Health (Ricerca Corrente); MLW was supported by Government funding of clinical research within NHS Sweden (ALF); KN was supported by Thure Carlsson Foundation; CN was supported by Swedish Alzheimer Fund. IRAM and GYRH were supported by CIHR (grant 74580) PARF (grant C06-01). JG was supported by the NINDS intramural research funds for FTD research. CMM was supported by Medical Research Council UK, Brains for Dementia Research, Alzheimer's Society, Alzheimer's Research UK, National Institutes for Health Research, Department of Health, Yvonne Mairy Bequest and acknowledges that tissue made available for this study was provided by the Newcastle Brain Tissue Resource, which was funded in part by grants G0400074 and G1100540 from the UK MRC, the Alzheimer's Research Trust and Alzheimer's Society through the Brains for Dementia Research Initiative and an NIHR Biomedical Research Centre Grant in Ageing and Health, and NIHR Biomedical Research Unit in Lewy Body Disorders. CMM was supported by the UK Department of Health and Medical Research Council and the Research was supported by the National Institute for Health Research Newcastle Biomedical Research Centre based at Newcastle Hospitals Foundation Trust and Newcastle University and acknowledges that the views expressed are those of the authors and not necessarily those of the NHS, the NIHR or the Department of Health; JA was supported by MRC, Dunhill Medical Trust, Alzheimer's Research UK; TDG was supported by Wellcome Trust Senior Clinical Fellow; IGM was supported by NIHR Biomedical Research Centre and Unit on Ageing Grants and acknowledges the National Institute for Health Research Newcastle Biomedical Research Centre based at Newcastle Hospitals Foundation Trust and Newcastle University. The views expressed are those of the author(s) and not necessarily those of the NHS, the NIHR or the Department of Health; AJT was supported by Medical Research Council, Alzheimer's Society, Alzheimer's Research UK, National Institutes for Health Research. EJ was supported by NIHR, Newcastle Biomedical Research Centre. PP, CR, SOC and EA were supported partially by FIMA (Foundation for Applied Medical Research); PP acknowledges Manuel Seijo-Martínez (Department of Neurology, Hospital do Salnés, Pontevedra, Spain), Ramon Rene, Jordi Gascon and Jaume Campdelacreu (Department of Neurology, Hospital de Bellvitge, Barcelona, Spain) for providing FTD DNA samples. RP, JDS, PA and AK were supported by German Federal Ministry of Education and Research (BMBF; grant number FKZ 01GI1007A – German FTLD consortium). IR was supported by Ministero dell'Istruzione, dell'Università e della Ricerca (MIUR) of Italy. PStGH was supported by the Canadian Institutes of Health Research, Wellcome Trust, Ontario Research Fund. FT was supported by the Italian Ministry of Health (ricerca corrente) and MIUR grant RBAP11FRE9; GR and GG were supported by the Italian Ministry of Health (ricerca corrente). JBR was supported by Camrbidge NIHR Biomedical Research Centre and Wellcome Trust (088324). JU, JC, SM were supported by the MRC Prion Unit core funding and acknowledge MRC UK, UCLH Biomedical Research Centre, Queen Square Dementia BRU; SM acknowledges the work of John Beck, Tracy Campbell, Gary Adamson, Ron Druyeh, Jessica Lowe, Mark Poulter. AD acknowledges the work of Benedikt Bader and of Manuela Neumann, Sigrun Roeber, Thomas Arzberger and Hans Kretzschmar†; VMVD and JQT were supported by Grants AG032953, AG017586 and AG010124; MG was supported by Grants AG032953, AG017586, AG010124 and NS044266; VMVD acknowledges EunRan Suh, PhD for assistance with sample handling and Elisabeth

McCarty-Wood for help in selection of cases; JQT acknowledges Terry Schuck, John Robinson and Kevin Raible for assistance with neuropathological evaluation of cases. CVB and the Antwerp site were in part funded by the MetLife Foundation for Medical Research Award (to CVB); the Belgian Science Policy Office (BELSPO) Interuniversity Attraction Poles program; the Flemish Government initiated Methusalem Excellence Program (to CVB); the Flemish government initiated Impulse Program on Networks for Dementia Research (VIND); the Research Foundation Flanders (FWO) and the University of Antwerp Research Fund. CVB and JvdZ acknowledge the neurologists S Engelborghs, PP De Deyn, A Sieben, R Vandenberghe and the neuropathologist JJ Martin for the clinical and pathological diagnoses. CVB and JvdZ further thank the personnel of the Neuromics Support Facility of the VIB Center for Molecular Neurology and the Antwerp Biobank of the Institute Born-Bunge for their expert support. IL and AB were supported by the program “Investissements d’avenir” ANR-10-IAIHU-06 and acknowledges the contribution of The French research network on FTLD/FTLD-ALS for the contribution in samples collection. BN is founded by Fondazione Cassa di Risparmio di Pistoia e Pescia (grant 2014.0365), SS is founded by the Cassa di Risparmio di Firenze (grant 2014.0310) and a grant from Ministry of Health n° RF-2010-2319722. JEN was supported by the Novo Nordisk Foundation, Denmark. MR was supported by the German National Genome Network (NGFN); German Ministry for Education and Research Grant Number 01GS0465. JDR, MNR, NCF and JDW were supported by an MRC programme grant and the Dementia Platform UK, the NIHR Queen Square Dementia Biomedical Research Unit (BRU) and the Leonard Wolfson Experimental Neurology Centre. MGS was supported by MRC grant n G0301152, Cambridge Biomedical Research Centre and acknowledges Mrs K Westmore for extracting DNA. HM was supported by the Motor Neuron Disease Association (Grant 6057). RR was supported by P50 AG016574, R01 NS080882, R01 NS065782, P50 NS72187 and the Consortium for Frontotemporal Dementia; DWD was supported by P50NS072187, P50AG016574, State of Florida Alzheimer Disease Initiative, & CurePSP, Inc.; NRGR, JEP, RCP, DK, BFB were supported by P50 AG016574; KAJ was supported by R01 AG037491; WWS was supported by NIH AG023501, AG019724, Consortium for Frontotemporal Dementia Research; BLM was supported by P50AG023501, P01AG019724, Consortium for FTD Research; HR was supported by AG032306. JCvS was supported by Stichting Dioraphte Foundation (11 02 03 00), Nuts Ohra Foundation (0801-69), Hersenstichting Nederland (BG 2010-02) and Alzheimer Nederland. CG and HHC acknowledge families, patients, clinicians including Dr Inger Nennesmo and Dr Vesna Jelic, Professor Laura Fratiglioni for control samples and Jenny Björkström, Håkan Thonberg, Charlotte Forsell, Anna-Karin Lindström and Lena Lilius for sample handling. CG was supported by Swedish Brain Power (SBP), the Strategic Research Programme in Neuroscience at Karolinska Institutet (StratNeuro), the regional agreement on medical training and clinical research (ALF) between Stockholm County Council and Karolinska Institutet, Swedish Alzheimer Foundation, Swedish Research Council, Karolinska Institutet PhD-student funding, King Gustaf V and Queen Victoria’s Free Mason Foundation. FP, AR, VD and FL acknowledge Labex DISTALZ. RF acknowledges the help and support of Mrs. June Howard at the Texas Tech University Health Sciences Center Office of Sponsored Programs for tremendous help in managing Material Transfer Agreement at TTUHSC.

APPENDIX B

IFGC members and affiliations

Raffaele Ferrari (UCL, Department of Molecular Neuroscience, Russell Square House, 9-12 Russell Square House, London, WC1B 5EH), Dena G Hernandez (Laboratory of Neurogenetics, National Institute on Aging, National Institutes of Health, Building 35, Room 1A215, 35 Convent Drive, Bethesda, MD 20892, USA; Reta Lila Weston Research Laboratories, Department of Molecular Neuroscience, UCL Institute of Neurology, London WC1N 3BG, UK), Michael A Nalls (Laboratory of Neurogenetics, National Institute on Aging, National Institutes of Health Building 35, Room 1A215, 35 Convent Drive, Bethesda, MD 20892, USA), Jonathan D Rohrer (Reta Lila Weston Research Laboratories, Department of Molecular Neuroscience, UCL Institute of Neurology, Queen Square, London WC1N 3BG, UK; Dementia Research Centre, Department of Neurodegenerative Disease, UCL Institute of Neurology), Adaikalavan Ramasamy (Reta Lila Weston Research Laboratories, Department of Molecular Neuroscience, UCL Institute of Neurology, London WC1N 3BG, UK; Department of Medical and Molecular Genetics, King's College London Tower Wing, Guy's Hospital, London SE1 9RT, UK; The Jenner Institute, University of Oxford, Roosevelt Drive, Oxford OX3 7BQ, UK), John BJ Kwok (Neuroscience Research Australia, Sydney, NSW 2031, Australia; School of Medical Sciences, University of New South Wales, Sydney, NSW 2052, Australia), Carol Dobson-Stone (Neuroscience Research Australia, Sydney, NSW 2031, Australia; School of Medical Sciences, University of New South Wales, Sydney, NSW 2052, Australia), William S Brooks Neuroscience Research Australia, Sydney, NSW 2031, Australia; Prince of Wales Clinical School, University of New South Wales, Sydney, NSW 2052, Australia), Peter R Schofield (Neuroscience Research Australia, Sydney, NSW 2031, Australia; School of Medical Sciences, University of New South Wales, Sydney, NSW 2052, Australia), Glenda M Halliday (Neuroscience Research Australia, Sydney, NSW 2031, Australia; School of Medical Sciences, University of New South Wales, Sydney, NSW 2052, Australia), John R Hodges (Neuroscience Research Australia, Sydney, NSW 2031, Australia; School of Medical Sciences, University of New South Wales, Sydney, NSW 2052, Australia), Olivier Piguet (Neuroscience Research Australia, Sydney, NSW 2031, Australia; School of Medical Sciences, University of New South Wales, Sydney, NSW 2052, Australia), Lauren Bartley (Neuroscience Research Australia, Sydney, NSW 2031, Australia), Elizabeth Thompson (South Australian Clinical Genetics Service, SA Pathology (at Women's and Children's Hospital), North Adelaide, SA 5006, Australia; Department of Paediatrics, University of Adelaide, Adelaide, SA 5000, Australia), Isabel Hernández (Research Center and Memory Clinic of Fundació ACE, Institut Català de Neurociències Aplicades, Barcelona, Spain), Agustín Ruiz (Research Center and Memory Clinic of Fundació ACE, Institut Català de Neurociències Aplicades, Barcelona, Spain), Mercè Boada (Research Center and Memory Clinic of Fundació ACE, Institut Català de Neurociències Aplicades, Barcelona, Spain), Barbara Borroni (Neurology Clinic, University of Brescia, Brescia, Italy), Alessandro Padovani (Neurology Clinic, University of Brescia, Brescia, Italy), Carlos Cruchaga (Department of Psychiatry, Washington University, St. Louis, MO, USA; Hope Center, Washington University School of Medicine, St. Louis, MO, USA), Nigel J Cairns (Hope Center, Washington University School of Medicine, St. Louis, MO, USA; Department of Pathology and Immunology, Washington University, St. Louis, MO, USA), Luisa Benussi (Molecular Markers Laboratory, IRCCS Istituto Centro San Giovanni di Dio Fatebenefratelli,Brescia, Italy), Giuliano Binetti (MAC Memory Clinic, IRCCS Istituto Centro San Giovanni di Dio Fatebenefratelli, Brescia, Italy), Roberta Ghidoni (Molecular Markers Laboratory, IRCCS Istituto Centro San Giovanni di Dio

Fatebenefratelli, Brescia, Italy), Gianluigi Forloni (Biology of Neurodegenerative Disorders, IRCCS Istituto di Ricerche Farmacologiche, "Mario Negri", Milano, Italy), Diego Albani (Biology of Neurodegenerative Disorders, IRCCS Istituto di Ricerche Farmacologiche "Mario Negri", Milano, Italy), Daniela Galimberti (University of Milan, Milan, Italy; Fondazione Cà Granda, IRCCS Ospedale Maggiore Policlinico, via F. Sforza 35, 20122, Milan, Italy), Chiara Fenoglio (University of Milan, Milan, Italy; Fondazione Cà Granda, IRCCS Ospedale Maggiore Policlinico, via F. Sforza 35, 20122, Milan, Italy), Maria Serpente (University of Milan, Milan, Italy; Fondazione Cà Granda, IRCCS Ospedale Maggiore Policlinico, via F. Sforza 35, 20122, Milan, Italy), Elio Scarpini (University of Milan, Milan, Italy; Fondazione Cà Granda, IRCCS Ospedale Maggiore Policlinico, via F. Sforza 35, 20122, Milan, Italy), Jordi Clarimón (Memory Unit, Neurology Department and Sant Pau Biomedical Research Institute, Hospital de la Santa Creu i Sant Pau, Universitat Autònoma de Barcelona, Barcelona, Spain; Center for Networker Biomedical Research in Neurodegenerative Diseases (CIBERNED), Madrid, Spain), Alberto Lleó (Memory Unit, Neurology Department and Sant Pau Biomedical Research Institute, Hospital de la Santa Creu i Sant Pau, Universitat Autònoma de Barcelona, Barcelona, Spain; Center for Networker Biomedical Research in Neurodegenerative Diseases (CIBERNED), Madrid, Spain), Rafael Blesa (Memory Unit, Neurology Department and Sant Pau Biomedical Research Institute, Hospital de la Santa Creu i Sant Pau, Universitat Autònoma de Barcelona, Barcelona, Spain; Center for Networker Biomedical Research in Neurodegenerative Diseases (CIBERNED), Madrid, Spain); Maria Landqvist Waldö (Unit of Geriatric Psychiatry, Department of Clinical Sciences, Lund University, Lund, Sweden), Karin Nilsson (Unit of Geriatric Psychiatry, Department of Clinical Sciences, Lund University, Lund, Sweden), Christer Nilsson (Clinical Memory Research Unit, Department of Clinical Sciences, Lund University, Lund, Sweden), Ian RA Mackenzie (Department of Pathology and Laboratory Medicine, University of British Columbia, Vancouver, Canada), Ging-Yuek R Hsiung (Division of Neurology, University of British Columbia, Vancouver, Canada), David MA Mann (Institute of Brain, Behaviour and Mental Health, University of Manchester, Salford Royal Hospital, Stott Lane, Salford, M6 8HD, UK), Jordan Grafman (Rehabilitation Institute of Chicago, Departments of Physical Medicine and Rehabilitation, Psychiatry, and Cognitive Neurology & Alzheimer's Disease Center; Feinberg School of Medicine, Northwestern University, Chicago, USA; Department of Psychology, Weinberg College of Arts and Sciences, Northwestern University, , Chicago, USA), Christopher M Morris (Newcastle Brain Tissue Resource, Institute for Ageing, Newcastle University, NE4 5PL, Newcastle upon Tyne, UK; Newcastle University, Institute of Neuroscience and Institute for Ageing, Campus for Ageing and Vitality, NE4 5PL, Newcastle upon Tyne, UK; Institute of Neuroscience, Newcastle University Medical School, Framlington Place NE2 4HH, Newcastle upon Tyne, UK), Johannes Attems (Newcastle University, Institute of Neuroscience and Institute for Ageing, Campus for Ageing and Vitality, NE4 5PL, Newcastle upon Tyne, UK; Timothy D Griffiths (Institute of Neuroscience, Newcastle University Medical School, Framlington Place, NE2 4HH, Newcastle upon Tyne, UK), Ian G McKeith (Newcastle University, Institute of Neuroscience and Institute for Ageing, Campus for Ageing and Vitality, Newcastle University, NE4 5PL, Newcastle upon Tyne, UK), Alan J Thomas (Newcastle University, Institute of Neuroscience and Institute for Ageing, Campus for Ageing and Vitality, NE4 5PL, Newcastle upon Tyne, UK), Pietro Pietrini (IMT School for Advanced Studies, Lucca, Lucca, Italy), Edward D Huey (Taub Institute, Departments of Psychiatry and Neurology, Columbia University, 630 West 168th Street New York, NY 10032), Eric M Wassermann (Behavioral Neurology Unit, National Insititute of Neurological Disorders and Stroke, National Insititutes of Health, 10 CENTER DR MSC 1440, Bethesda, MD 20892-1440), Atik Baborie (Department of Laboratory Medicine & Pathology, Walter Mackenzie Health Sciences Centre, 8440 - 112 St, University of Alberta Edmonton, Alberta T6G 2B7, Canada), Evelyn Jaros (Newcastle University, Institute for Ageing and Health, Campus for Ageing and Vitality, NE4 5PL, Newcastle upon Tyne, UK), Michael C Tierney (Behavioral Neurology Unit, National

Insititute of Neurological Disorders and Stroke, National Insititutes of Health, 10 CENTER DR MSC 1440, Bethesda, MD 20892-1440), Pau Pastor (Center for Networker Biomedical Research in Neurodegenerative Diseases (CIBERNED), Madrid, Spain; Neurogenetics Laboratory, Division of Neurosciences, Center for Applied Medical Research, Universidad de Navarra, Pamplona, Spain; Department of Neurology, Clínica Universidad de Navarra, University of Navarra School of Medicine, Pamplona, Spain), Cristina Razquin (Neurogenetics Laboratory, Division of Neurosciences, Center for Applied Medical Research, Universidad de Navarra, Pamplona, Spain), Sara Ortega-Cubero (Center for Networker Biomedical Research in Neurodegenerative Diseases (CIBERNED), Madrid, Spain; Neurogenetics Laboratory, Division of Neurosciences, Center for Applied Medical Research, Universidad de Navarra, Pamplona, Spain), Elena Alonso (Neurogenetics Laboratory, Division of Neurosciences, Center for Applied Medical Research, Universidad de Navarra, Pamplona, Spain), Robert Perneczky (Neuroepidemiology and Ageing Research Unit, School of Public Health, Faculty of Medicine, The Imperial College of Science, Technology and Medicine, London W6 8RP, UK; West London Cognitive Disorders Treatment and Research Unit, West London Mental Health Trust, London TW8 8 DS, UK; Department of Psychiatry and Psychotherapy, Technische Universität München, Munich, 81675 Germany), Janine Diehl-Schmid (Department of Psychiatry and Psychotherapy, Technische Universität München, Munich, 81675 Germany), Panagiotis Alexopoulos (Department of Psychiatry and Psychotherapy, Technische Universität München, Munich, 81675 Germany), Alexander Kurz (Department of Psychiatry and Psychotherapy, Technische Universität München, Munich, 81675 Germany), Innocenzo Rainero (Neurology I, Department of Neuroscience, University of Torino, Italy, A.O. Città della Salute e della Scienza di Torino, Torino, Italy), Elisa Rubino (Neurology I, Department of Neuroscience, University of Torino, Italy, A.O. Città della Salute e della Scienza di Torino, Torino, Italy), Lorenzo Pinessi (Neurology I, Department of Neuroscience, University of Torino, Italy, A.O. Città della Salute e della Scienza di Torino, Torino, Italy), Ekaterina Rogaeva (Tanz Centre for Research in Neurodegenerative Diseases, University of Toronto 60 Leonard Street, Toronto, Ontario, Canada, M5T 2S8), Peter St George-Hyslop(Tanz Centre for Research in Neurodegenerative Diseases, University of Toronto, 60 Leonard Street, Toronto, Ontario, Canada, M5T 2S8; Cambridge Institute for Medical Research, and the Department of Clinical Neurosciences, University of Cambridge, Hills Road, Cambridge, UK CB2 0XY), Giacomina Rossi (Division of Neurology V and Neuropathology, Fondazione IRCCS Istituto Neurologico Carlo Besta, 20133 Milano, Italy), Fabrizio Tagliavini (Division of Neurology V and Neuropathology, Fondazione IRCCS Istituto Neurologico Carlo Besta, 20133 Milano, Italy), Giorgio Giaccone (Division of Neurology V and Neuropathology, Fondazione IRCCS Istituto Neurologico Carlo Besta, 20133 Milano, Italy), James B Rowe Cambridge University Department of Clinical Neurosciences, Cambridge, CB2 0SZ, UK; MRC Cognition and Brain Sciences Unit, Cambridge, CB2 7EF, UK; Behavioural and Clinical Neuroscience Institute, Cambridge, CB2 3EB, UK), Johannes CM Schlachetzki (University of California San Diego, Department of Cellular & Molecular Medicine, 9500 Gilman Drive, La Jolla, CA, 92093), James Uphill (MRC Prion Unit, Department of Neurodegenerative Disease, UCL Institute of Neurology, Queen Square House, Queen Square, London, WC1N 3BG), John Collinge (MRC Prion Unit, Department of Neurodegenerative Disease, UCL Institute of Neurology Queen Square House, Queen Square, London, WC1N 3BG), Simon Mead (MRC Prion Unit, Department of Neurodegenerative Disease, UCL Institute of Neurology, Queen Square House, Queen Square, London, WC1N 3BG), Adrian Danek (Neurologische Klinik und Poliklinik, Ludwig-Maximilians-Universität, Munich, Germany, German Center for Neurodegenerative Diseases (DZNE)), Vivianna M Van Deerlin (University of Pennsylvania Perelman School of Medicine, Department of Pathology and Laboratory Medicine, Philadelphia, PA, USA), Murray Grossman (University of Pennsylvania Perelman School of Medicine, Department of Neurology and Penn Frontotemporal Degeneration Center, Philadelphia, PA, USA), John Q Trojanowski (University of Pennsylvania Perelman School of

Medicine, Department of Pathology and Laboratory Medicine, Philadelphia, PA, USA), Julie van der Zee (Neurodegenerative Brain Diseases group, VIB-UAntwerp Center of Molecular Neurology, Antwerp, Belgium; Laboratory of Neurogenetics, Institute Born-Bunge, University of Antwerp, Antwerp, Belgium), Christine Van Broeckhoven (Neurodegenerative Brain Diseases group, VIB-UAntwerp Center of Molecular Neurology, Antwerp, Belgium; Laboratory of Neurogenetics, Institute Born-Bunge, University of Antwerp, Antwerp, Belgium), Stefano F Cappa (Neurorehabilitation Unit, Dept. Of Clinical Neuroscience, Vita-Salute University and San Raffaele Scientific Institute, Milan, Italy), Isabelle Leber (Inserm, UMR_S975, CRICM; UPMC Univ Paris 06, UMR_S975; CNRS UMR 7225, F-75013, Paris, France; AP-HP, Hôpital de la Salpêtrière, Département de neurologie-centre de références des démences rares, F-75013, Paris, France), Didier Hannequin (Service de Neurologie, Inserm U1079, CNR-MAJ, Rouen University Hospital, Rouen, France), Véronique Golfier (Service de neurologie, CH Saint Brieuc, France), Martine Vercelletto (Service de neurologie, CHU Nantes, France), Alexis Brice (Inserm, UMR_S975, CRICM; UPMC Univ Paris 06, UMR_S975; CNRS UMR 7225, F-75013, Paris, France; AP-HP, Hôpital de la Salpêtrière, Département de neurologie-centre de références des démences rares, F-75013, Paris, France), Benedetta Nacmias (Department of Neurosciences, Psychology, Drug Research and Child Health (NEUROFARBA) University of Florence, Florence, Italy), Sandro Sorbi (Department of Neurosciences, Psychology, Drug Research and Child Health (NEUROFARBA), University of Florence and IRCCS "Don Carlo Gnocchi" Firenze, Florence, Italy), Silvia Bagnoli (Department of Neurosciences, Psychology, Drug Research and Child Health (NEUROFARBA), University of Florence, Florence, Italy), Irene Piaceri (Department of Neurosciences, Psychology, Drug Research and Child Health (NEUROFARBA), University of Florence, Florence, Italy), Jørgen E Nielsen (Danish Dementia Research Centre, Neurogenetics Clinic, Department of Neurology, Rigshospitalet, Copenhagen University Hospital, Copenhagen, Denmark; Department of Cellular and Molecular Medicine, Section of Neurogenetics, The Panum Institute, University of Copenhagen, Copenhagen, Denmark), Lena E Hjermind (Danish Dementia Research Centre, Neurogenetics Clinic, Department of Neurology, Rigshospitalet, Copenhagen University Hospital, Copenhagen, Denmark; Department of Cellular and Molecular Medicine, Section of Neurogenetics, The Panum Institute, University of Copenhagen, Copenhagen, Denmark), Matthias Riemenschneider (Saarland University Hospital, Department for Psychiatry & Psychotherapy, Kirrberger Str.1, Bld.90, 66421 Homburg/Saar, Germany; Saarland University, Laboratory for Neurogenetics, Kirrberger Str.1, Bld.90, 66421 Homburg/Saar, Germany), Manuel Mayhaus (Saarland University, Laboratory for Neurogenetics, Kirrberger Str.1, Bld.90, 66421 Homburg/Saar, Germany), Bernd Ibach (University Regensburg, Department of Psychiatry, Psychotherapy and Psychosomatics, Universitätsstr. 84, 93053 Regensburg, Germany), Gilles Gasparoni (Saarland University, Laboratory for Neurogenetics, Kirrberger Str.1, Bld.90, 66421 Homburg/Saar, Germany), Sabrina Pichler (Saarland University, Laboratory for Neurogenetics, Kirrberger Str.1, Bld.90, 66421 Homburg/Saar, Germany), Wei Gu (Saarland University, Laboratory for Neurogenetics, Kirrberger Str.1, Bld.90, 66421 Homburg/Saar, Germany; Luxembourg Centre For Systems Biomedicine (LCSB), University of Luxembourg 7, avenue des Hauts-Fourneaux, 4362 Esch-sur-Alzette, Luxembourg), Martin N Rossor (Dementia Research Centre, Department of Neurodegenerative Disease, UCL Institute of Neurology, Queen Square, London, WC1N 3BG), Nick C Fox (Dementia Research Centre, Department of Neurodegenerative Disease, UCL Institute of Neurology Queen Square, London, WC1N 3BG), Jason D Warren (Dementia Research Centre, Department of Neurodegenerative Disease, UCL Institute of Neurology, Queen Square, London, WC1N 3BG), Maria Grazia Spillantini (University of Cambridge, Department of Clinical Neurosciences, John Van Geest Brain Repair Centre, Forvie Site, Robinson way, Cambridge CB2 0PY), Huw R Morris (UCL, Department of Molecular Neuroscience, Russell Square House, 9-12 Russell Square House, London, WC1B 5EH), Patrizia Rizzu (German Center for Neurodegenerative Diseases-Tübingen, Otfried

Muellerstrasse 23, Tuebingen 72076, Germany), Peter Heutink (German Center for Neurodegenerative Diseases-Tübingen, Otfried Muellerstrasse 23, Tuebingen 72076, Germany), Julie S Snowden (Institute of Brain, Behaviour and Mental Health, Faculty of Medical and Human Sciences, University of Manchester, Manchester, UK), Sara Rollinson (Institute of Brain, Behaviour and Mental Health, Faculty of Medical and Human Sciences, University of Manchester, Manchester, UK), Anna Richardson (Salford Royal Foundation Trust, Faculty of Medical and Human Sciences, University of Manchester, Manchester, UK), Alexander Gerhard (Institute of Brain, Behaviour and Mental Health, The University of Manchester, 27 Palatine Road, Withington, Manchester, M20 3LJ, UK), Amalia C Bruni (Regional Neurogenetic Centre, ASPCZ, Lamezia Terme, Italy), Raffaele Maletta (Regional Neurogenetic Centre, ASPCZ, Lamezia Terme, Italy), Francesca Frangipane (Regional Neurogenetic Centre, ASPCZ, Lamezia Terme, Italy), Chiara Cupidi (Regional Neurogenetic Centre, ASPCZ, Lamezia Terme, Italy), Livia Bernardi (Regional Neurogenetic Centre, ASPCZ, Lamezia Terme, Italy), Maria Anfossi (Regional Neurogenetic Centre, ASPCZ, Lamezia Terme, Italy), Maura Gallo (Regional Neurogenetic Centre, ASPCZ, Lamezia Terme, Italy), Maria Elena Conidi Regional Neurogenetic Centre, ASPCZ, Lamezia Terme, Italy), Nicoletta Smirne (Regional Neurogenetic Centre, ASPCZ, Lamezia Terme, Italy), Rosa Rademakers (Department of Neuroscience, Mayo Clinic Jacksonville, 4500 San Pablo Road, Jacksonville, FL 32224), Matt Baker (Department of Neuroscience, Mayo Clinic Jacksonville, 4500 San Pablo Road, Jacksonville, FL 32224), Dennis W Dickson (Department of Neuroscience, Mayo Clinic Jacksonville, 4500 San Pablo Road, Jacksonville, FL 32224), Neill R Graff-Radford (Department of Neurology, Mayo Clinic Jacksonville, 4500 San Pablo Road, Jacksonville, FL 32224), Ronald C Petersen (Department of Neurology, Mayo Clinic Rochester, 2001st street SW Rochester MN 5905), David Knopman (Department of Neurology, Mayo Clinic Rochester, 2001st street SW Rochester MN 5905), Keith A Josephs (Department of Neurology, Mayo Clinic Rochester, 2001st street SW Rochester MN 5905), Bradley F Boeve (Department of Neurology, Mayo Clinic Rochester, 2001st street SW Rochester MN 5905), Joseph E Parisi (Department of Pathology, Mayo Clinic Rochester, 2001st street SW Rochester MN 5905), William W Seeley (Department of Neurology, Box 1207, University of California, San Francisco, CA 94143, USA), Bruce L Miller (Memory and Aging Center, Department of Neurology, University of California, San Francisco, CA 94158, USA), Anna M Karydas (Memory and Aging Center, Department of Neurology, University of California, San Francisco, CA 94158, USA), Howard Rosen (Memory and Aging Center, Department of Neurology, University of California, San Francisco, CA 94158, USA), John C van Swieten (Department of Neurology, Erasmus Medical Centre, Rotterdam, The Netherlands; Department of Medical Genetics, VU university Medical Centre, Amsterdam, The Netherlands), Elise GP Dopper (Department of Neurology, Erasmus Medical Centre, Rotterdam, The Netherlands), Harro Seelaar (Department of Neurology, Erasmus Medical Centre, Rotterdam, The Netherlands), Yolande AL Pijnenburg (Alzheimer Centre and department of neurology, VU University medical centre, Amsterdam, The Netherlands), Philip Scheltens (Alzheimer Centre and department of neurology, VU University medical centre, Amsterdam, The Netherlands), Giancarlo Logroscino (Department of Basic Medical Sciences, Neurosciences and Sense Organs of the "Aldo Moro" University of Bari, Bari, Italy), Rosa Capozzo (Department of Basic Medical Sciences, Neurosciences and Sense Organs of the "Aldo Moro" University of Bari, Bari, Italy), Valeria Novelli (Medical Genetics Unit, Fondazione Policlinico Universitario A. Gemelli, Rome, Italy), Annibale A Puca (Cardiovascular Research Unit, IRCCS Multimedica, Milan, Italy; Department of Medicine and Surgery, University of Salerno, Baronissi (SA), Italy), Massimo Franceschi (Neurology Dept, IRCCS Multimedica, Milan, Italy), Alfredo Postiglione (Department of Clinical Medicine and Surgery, University of Naples Federico II, Naples, Italy), Graziella Milan (Geriatric Center Frullone- ASL Napoli 1 Centro, Naples, Italy), Paolo Sorrentino (Geriatric Center Frullone- ASL Napoli 1 Centro, Naples, Italy), Mark Kristiansen (UCL Genomics, Institute of Child Health (ICH), UCL, London, UK), Huei-Hsin Chiang (Karolinska

Institutet, Dept NVS, Alzheimer Research Center, Novum, SE-141 57, Stockholm, Sweden; Dept of Geriatric Medicine, Genetics Unit, M51, Karolinska University Hospital, SE-14186, Stockholm), Caroline Graff (Karolinska Institutet, Dept NVS, Alzheimer Research Center, Novum, SE-141 57, Stockholm, Sweden; Dept of Geriatric Medicine, Genetics Unit, M51, Karolinska University Hospital, SE-14186, Stockholm), Florence Pasquier (Univ Lille, Inserm 1171, DISTALZ, CHU 59000 Lille, France), Adeline Rollin (Univ Lille, Inserm 1171, DISTALZ, CHU 59000 Lille, France), Vincent Deramecourt (Univ Lille, Inserm 1171, DISTALZ, CHU 59000 Lille, France), Thibaud Lebouvier (Univ Lille, Inserm 1171, DISTALZ, CHU 59000 Lille, France), Dimitrios Kapogiannis (National Institute on Aging (NIA/NIH), 3001 S. Hanover St, NM 531, Baltimore, MD, 21230), Luigi Ferrucci (Clinical Research Branch, National Institute on Aging, Baltimore, MD, USA), Stuart Pickering-Brown (Institute of Brain, Behaviour and Mental Health, Faculty of Medical and Human Sciences, University of Manchester, Manchester, UK), Andrew B Singleton (Laboratory of Neurogenetics, National Institute on Aging, National Institutes of Health, Building 35, Room 1A215, 35 Convent Drive, Bethesda, MD 20892, USA), John Hardy (UCL, Department of Molecular Neuroscience, Russell Square House, 9-12 Russell Square House, London, WC1B 5EH), Parastoo Momeni (Laboratory of Neurogenetics, Department of Internal Medicine, Texas Tech University Health Science Center, 4th street, Lubbock, Texas 79430, USA)

R Ferrari; D G Hernandez; M A Nalls; J D Rohrer; A Ramasamy; J B J Kwok; C Dobson-Stone; P R Schofield; G M Halliday; J R Hodges; O Piguet; L Bartley; E Thompson; I Hernández; A Ruiz; M Boada; B Borroni; A Padovani; C Cruchaga; N J Cairns; L Benussi; G Binetti; R Ghidoni; G Forloni; D Albani; D Galimberti; C Fenoglio; M Serpente; E Scarpini; J Clarimón; A Lleó; R Blesa; M Landqvist Waldö; K Nilsson; C Nilsson; I R A Mackenzie; G-Y R Hsiung; D M A Mann; J Grafman; C M Morris; J Attems; T D Griffiths; I G McKeith; A J Thomas; P Pietrini; E D Huey; E M Wassermann; A Baborie; E Jaros; M C Tierney; P Pastor; C Razquin; S Ortega-Cubero; E Alonso; R Perneczky; J Diehl-Schmid; P Alexopoulos; A Kurz; I Rainero; E Rubino; L Pinessi; E Rogaeva; P St George-Hyslop; G Rossi; F Tagliavini; G Giaccone; D. Albani; J B Rowe; J C M Schlachetzki; J Uphill; J Collinge; S Mead; A Danek; V M Van Deerlin; M Grossman; J Q Trojanowski; J van der Zee; C Van Broeckhoven; S F Cappa; I Leber; D Hannequin; V Golfier; M Vercelletto; A Brice; B Nacmias; S Sorbi; S Bagnoli; I Piaceri; J E Nielsen; L E Hjermind; M Riemenschneider; M Mayhaus; B Ibach; G Gasparoni; S Pichler; W Gu; M N Rossor; N C Fox; J D Warren; M G Spillantini; H R Morris; P Rizzu; P Heutink; J S Snowden; S Rollinson; A Richardson; A Gerhard; A C Bruni; R Maletta; F Frangipane; C Cupidi; L Bernardi; M Anfossi; M Gallo; M E Conidi; N Smirne; R Rademakers; M Baker; D W Dickson; N R Graff-Radford; R C Petersen; D Knopman; K A Josephs; B F Boeve; J E Parisi; W W Seeley; B L Miller; A M Karydas; H Rosen; J C van Swieten; E G P Dopper; H Seelaar; Y A L Pijnenburg; P Scheltens; G Logroscino; R Capozzo; V Novelli; A A Puca; M Franceschi; A Postiglione; G Milan; P Sorrentino; M Kristiansen; H-H Chiang; C Graff; F Pasquier; A Rollin; V Deramecourt; T Lebouvier; D Kapogiannis; L Ferrucci; S Pickering-Brown; A B Singleton; J Hardy; P Momeni

Raffaele Ferrari, Dena G Hernandez, Michael A Nalls, Jonathan D Rohrer, Adaikalavan Ramasamy, John BJ Kwok, Carol Dobson-Stone, William S Brooks, Peter R Schofield, Glenda M Halliday, John R Hodges, Olivier Piguet, Lauren Bartley, Elizabeth Thompson, Isabel Hernández, Agustín Ruiz, Mercè Boada, Barbara Borroni, Alessandro Padovani, Carlos Cruchaga, Nigel J Cairns, Luisa Benussi, Giuliano Binetti, Roberta Ghidoni, Gianluigi Forloni, Daniela Galimberti, Chiara Fenoglio, Maria Serpente, Elio Scarpini, Jordi Clarimón, Alberto Lleó, Rafael Blesa, Maria Landqvist Waldö, Karin Nilsson, Christer Nilsson, Ian RA Mackenzie, Ging-Yuek R Hsiung, David MA Mann, Jordan Grafman, Christopher M Morris, Johannes Attems, Timothy D Griffiths, Ian G McKeith, Alan J Thomas, Pietro Pietrini, Edward D Huey, Eric M Wassermann, Atik Baborie, Evelyn Jaros, Michael C Tierney, Pau Pastor, Cristina Razquin, Sara Ortega-Cubero, Elena Alonso, Robert Perneczky, Janine Diehl-Schmid, Panagiotis Alexopoulos, Alexander Kurz, Innocenzo Rainero, Elisa Rubino, Lorenzo Pinessi, Ekaterina Rogaeva, Peter St George-Hyslop, Giacomina Rossi, Fabrizio Tagliavini, Giorgio Giaccone, James B Rowe, Johannes CM Schlachetzki, James Uphill, John Collinge, Simon Mead, Adrian Danek, Vivianna M Van Deerlin, Murray Grossman, John Q Trojanowski, Julie van der Zee, Christine Van Broeckhoven, Stefano F Cappa, Isabelle Le Ber, Didier Hannequin, Véronique Golfier, Martine Vercelletto, Alexis Brice, Benedetta Nacmias, Sandro Sorbi, Silvia Bagnoli, Irene Piaceri, Jørgen E Nielsen, Lena E Hjermind, Matthias Riemenschneider, Manuel Mayhaus, Bernd Ibach, Gilles Gasparoni, Sabrina Pichler, Wei Gu, Martin N Rossor, Nick C Fox, Jason D Warren, Maria Grazia Spillantini, Huw R Morris, Patrizia Rizzu, Peter Heutink, Julie S Snowden, Sara Rollinson, Anna Richardson, Alexander Gerhard, Amalia C Bruni, Raffaele Maletta, Francesca Frangipane, Chiara Cupidi, Livia Bernardi, Maria Anfossi, Maura Gallo, Maria Elena Conidi, Nicoletta Smirne, Rosa Rademakers, Matt Baker, Dennis W Dickson, Neill R Graff-Radford, Ronald C Petersen, David Knopman, Keith A Josephs, Bradley F Boeve, Joseph E Parisi, William W Seeley, Bruce L Miller, Anna M Karydas, Howard Rosen, John C van Swieten, Elise GP Dopper, Harro Seelaar, Yolande AL Pijnenburg, Philip Scheltens, Giancarlo Logroscino, Rosa Capozzo, Valeria Novelli, Annibale A Puca, Massimo Franceschi, Alfredo Postiglione, Graziella Milan, Paolo Sorrentino, Mark Kristiansen, Huei-Hsin Chiang, Caroline Graff, Florence Pasquier, Adeline Rollin, Vincent Deramecourt, Florence Lebert, Dimitrios Kapogiannis, Luigi Ferrucci, Stuart Pickering-Brown, Andrew B Singleton, John Hardy and Parastoo Momeni.
